# Cardiologists’ perspectives on pharmacogenomics implementation in a hybrid health system: A qualitative study from the United Arab Emirates

**DOI:** 10.1371/journal.pone.0351218

**Published:** 2026-06-12

**Authors:** Maram O. Abbas, Azhar T. Rahma, Iffat Elbarazi, Bassam R. Ali, George P. Patrinos, Hana Ghadibah, Amna Al Muaini, Fatma Al-Maskari

**Affiliations:** 1 Institute of Public Health, College of Medicine and Health Sciences, United Arab Emirates University, Al Ain, United Arab Emirates; 2 Department of Genetics and Genomics, College of Medicine and Health Sciences, United Arab Emirates University, Al Ain, United Arab Emirates; 3 Zayed Centre for Health Sciences, United Arab Emirates University, Al Ain, United Arab Emirates; 4 Department of Pharmacy, School of Health Sciences, University of Patras, Patras, Greece; 5 Independent Researcher, Dubai, United Arab Emirates; 6 Tawam Hospital, Pharmacy Department, AL-Ain, United Arab Emirates; Northern Border University, SAUDI ARABIA

## Abstract

**Background:**

Pharmacogenomics (PGx) can optimise cardiovascular therapy, yet routine integration in cardiology remains limited. In the United Arab Emirates, a hybrid public–private health system, the real-world PGx use is still emerging. However, there is limited understanding of how cardiologists perceive and navigate PGx implementation within such complex health system contexts.

**Objective:**

To examine cardiologists’ perspectives on the feasibility, barriers, and facilitators of implementing PGx using the Consolidated Framework for Implementation Research (CFIR).

**Methods:**

A qualitative study using an abductive analytical approach was conducted through semi-structured interviews with 15 cardiologists from public and private institutions. Participants were recruited via purposive, convenience, and snowball sampling. Interviews were transcribed verbatim and thematically analysed in NVivo. The CFIR guided the analysis across intervention characteristics, outer setting, inner setting, individual characteristics, and process.

**Results:**

Clinicians expressed strong conceptual support for PGx, especially in higher-risk scenarios, but reported limited hands-on exposure and confidence. Barriers included perceived test complexity, cost, and lack of reimbursement; insufficient laboratory capacity and EHR integration; unclear workflows and role ownership; and turnaround times misaligned with acute care. Outer-setting constraints (ambiguous policy signals and payer criteria) and inner-setting variability (resources, leadership engagement, and communication pathways) further limited uptake. Reported facilitators included multidisciplinary service models (with input from pharmacists and genetics), targeted case-based training, initial deployment in non-acute contexts, and the structured capture of results with EHR-embedded clinical decision support.

**Conclusions:**

PGx implementation in cardiology within the UAE is shaped by structural, organisational, and workforce-level gaps. Addressing these through targeted clinical guidance, improved training, stronger reimbursement mechanisms, enhanced laboratory capacity, and integrated digital decision support may enable more equitable and scalable adoption. These findings provide actionable insights for health systems seeking to operationalise PGx within diverse or hybrid healthcare contexts.

## Introduction

Pharmacogenomics (PGx) investigates how genetic variation affects an individual’s response to medications, enabling personalised pharmacological therapy that optimises efficacy and minimises adverse effects [1,2]. As a central component of precision medicine, its clinical translation depends not only on scientific evidence but also on supportive health-system infrastructure and policy frameworks.

In cardiology, PGx is particularly relevant due to the widespread use of medications with narrow therapeutic indices and variable patient responses [3,4]. PGx has been increasingly applied in this field, where commonly prescribed drugs such as clopidogrel, warfarin, and statins demonstrate well-established gene–drug interactions. For example, CYP2C19 variants affect clopidogrel responsiveness and cardiovascular outcomes, CYP2C9 and VKORC1 variants influence warfarin dosing and bleeding risk, and SLCO1B1 variants are associated with statin-induced myopathy. Emerging evidence also suggests that ADRB1 variants may modulate response to beta-blockers. These gene–drug interactions have important clinical implications, contributing to variability in treatment response, risk of adverse drug reactions, and overall therapeutic outcomes in cardiovascular care [5–7].

To support the clinical application of this evidence, international guideline bodies, including the Clinical Pharmacogenetics Implementation Consortium (CPIC), the Dutch Pharmacogenetics Working Group (DPWG), the U.S. Food and Drug Administration (FDA), and the European Medicines Agency (EMA), provide evidence-based recommendations and drug-labelling guidance to support PGx-informed prescribing [8–11]. Despite this strong evidence base, integration of PGx into routine cardiovascular care remains inconsistent, with adoption largely constrained by health-system and organisational factors rather than gaps in clinical knowledge [12].

Globally, the determinants of PGx adoption in routine practice extend beyond clinical awareness and include systems-level challenges, such as limited health policy signals, inadequate reimbursement/insurance coverage, and insufficient IT infrastructure to support clinical decision-making [13–15]. These translational barriers are particularly relevant in hybrid health systems such as the United Arab Emirates (UAE), a high-income context characterised by rapidly developing infrastructure, diverse patient populations, and a mixed public–private funding model [16].

In the context of the UAE, previous studies have shown that while healthcare providers recognise the potential of PGx, its clinical use is limited by barriers such as a lack of education, inconsistent access to testing, and policy gaps [17–20]. Genomic studies have further highlighted that a significant proportion of UAE nationals and residents could benefit from PGx testing, given the high prevalence of PGx biomarkers relevant to commonly prescribed cardiovascular medications [21–24]. In response, the UAE has initiated early efforts to institutionalise PGx, exemplified by technical guidelines from the Department of Health Abu Dhabi recommending PGx testing for medications such as clopidogrel, warfarin, and statins [25]. Nevertheless, the real-world readiness of cardiologists, key prescribers of PGx-relevant therapies, to implement PGx in routine practice remains unclear.

Understanding cardiologists’ perspectives is crucial for health-system planning, given that frontline clinicians play a central role in adopting and sustaining precision-medicine interventions. Qualitative approaches are well-suited to exploring the complex interplay of individual, organisational, and policy-level determinants that shape PGx implementation. The Consolidated Framework for Implementation Research (CFIR) offers a structured approach to examining these multi-level determinants and has been widely applied in genomic and pharmacogenomic research [26,27]. While CFIR has been used to explore barriers and facilitators of PGx implementation across diverse international settings, context-specific qualitative evidence in cardiology within the UAE remains limited [28].

This study, therefore, applied the CFIR to examine cardiologists’ perceptions and real-world experiences with PGx implementation in the UAE. The aim is to identify context-specific barriers and facilitators to inform health-system strategies for integrating PGx into routine cardiovascular care.

## Methods

### Study design and setting

This study adopted a qualitative design with a descriptive orientation using an abductive analytical approach. Data were analysed inductively using thematic analysis to identify emerging themes, and the findings were subsequently mapped onto the Consolidated Framework for Implementation Research (CFIR) to provide a structured interpretation.

Semi-structured interviews were conducted to explore cardiologists’ perspectives on the implementation of PGx testing in cardiovascular care. The study was designed to generate in-depth insights into perceived barriers, facilitators, and system-level dynamics influencing the adoption of PGx in clinical cardiology. Data collection and reporting adhered to the Consolidated Criteria for Reporting Qualitative Research (COREQ) checklist to ensure methodological rigour, transparency, and credibility [29] (Table S1 in S1 File).

The study was situated within the UAE healthcare system, which has witnessed rapid transformation through health reforms aimed at improving clinical service quality, expanding infrastructure, and promoting innovation. The UAE operates a mandatory private health insurance system, supported by both government-funded and private-sector healthcare providers. This dual-system structure plays a crucial role in shaping clinical decision-making, reimbursement practices, and the integration of emerging technologies, such as PGx [30,31].

### Study tool: Development and validation

A semi-structured interview guide was designed to elicit rich, experience-based narratives while allowing participants to introduce ideas, reflect on personal clinical experiences, and highlight contextual factors beyond the scripted questions (Table S2 in S1 File).

A comprehensive literature review informed the guide on PGx adoption in clinical practice, and several question areas were adapted from previous qualitative studies on genomic medicine and health system implementation [32–34] The guide underwent expert review by PGx researchers and implementation science professionals, both locally and internationally, to ensure conceptual clarity and contextual relevance.

Although the guide was not explicitly structured around all CFIR constructs, its content was broadly aligned with its domains. This alignment supported the later application of CFIR as an analytical framework during data analysis and mapping of emergent themes.

The guide was piloted with two clinicians to refine the question flow and ensure clarity. It was then used consistently across interviews while allowing for probing and adaptation depending on participants’ responses.

### Sample size and sampling

A total of 15 cardiologists were recruited using a combined purposive, convenience, and snowball sampling strategy. Eligible participants were adult cardiologists practising in general cardiology, heart failure, or congenital heart disease across the UAE. Inclusion criteria focused on those who actively prescribed medications commonly associated with PGx testing (e.g., antiplatelets, anticoagulants, statins) and who were involved in clinical decision-making or treatment protocol development.

Recruitment began through two major hospital administrations in different Emirates, which shared study invitations with eligible cardiologists on behalf of the research team. Those who chose to participate often suggested colleagues who might offer useful perspectives, thereby expanding the sample through professional networks. Sampling combined purposive and convenience strategies to include cardiologists with prescribing roles and direct experience in PGx-related care. The team also aimed for diversity in career stage, from early-career specialists to senior consultants and department heads, and in workplace setting, with participants representing both public and private institutions across Abu Dhabi, Dubai, Sharjah, and the northern Emirates.

A total of 20 cardiologists were invited; 15 agreed to participate and completed the interviews, while five declined due to time constraints or unfamiliarity with PGx. Sample size was guided by data saturation, with interviews continuing until no new codes or themes were identified. Saturation was assessed through concurrent coding, iterative comparison of emerging themes, and regular team discussions. No new themes were identified in the final interviews, indicating that code saturation had been achieved [35].

### Data collection

Data were collected between November 2024 and February 2025. All interviews were conducted virtually via Microsoft Teams, audio-recorded with the participant’s consent, and transcribed verbatim by two independent investigators to ensure accuracy and minimise transcription bias.

All interviews were conducted by a female PhD candidate in Public Health, with a background in clinical pharmacy and formal training in qualitative research. She had no prior relationship with any of the participants.

Participants were initially invited via personalised emails, which included an information sheet and consent form. Reminder messages were sent one day and one hour before the scheduled interview to confirm attendance. Additionally, participants were sent the interview guide at least 48 hours in advance to allow them to reflect on the key discussion areas and prepare any comments or questions. The decision to share the interview guide in advance was intended to support more considered and practice-based responses, allowing participants to reflect on their real-world clinical experiences. Given the specialised and emerging nature of PGx, this approach helped ensure that responses were structured, relevant, and grounded in actual practice rather than being purely spontaneous or speculative. The interviews ranged from 30 to 60 minutes, depending on participant engagement and availability.

### Theoretical framework and data analysis

A thematic analysis explored cardiologists’ perspectives on integrating PGx into routine practice. Using an abductive approach, data-driven codes were developed inductively from interview transcripts and iteratively interpreted through established implementation frameworks.

The transcripts were initially processed using an AI-based tool to assist in identifying preliminary codes and themes. The tool was used solely for initial code suggestions; all outputs were critically reviewed, refined, and manually validated by the research team to ensure accuracy and relevance.

To enhance analytic rigour, a subset of transcripts was independently coded by two researchers. Coding discrepancies were discussed and resolved through consensus during regular team meetings. In line with qualitative research standards, formal statistical measures of intercoder reliability were not applied; instead, emphasis was placed on consistency, reflexive dialogue, and agreement in interpretation. An audit trail of coding and analytic decisions was maintained throughout the process.

Reflexivity was embedded throughout the analysis to enhance transparency and minimise interpretive bias. The researcher’s positionality was explicitly considered, and the multidisciplinary research team, bringing expertise in public health, pharmacogenomics, and implementation science, contributed diverse perspectives to data interpretation. Reflexive journaling documented methodological decisions, evolving interpretations, and underlying assumptions, while regular peer debriefings supported critical reflection and consistency in analysis.

To ensure the trustworthiness of the findings, multiple strategies were employed. Credibility was enhanced through member checking with a subset of participants and sustained engagement with the data. Dependability was supported through the audit trail and ongoing team discussions. Confirmability was ensured through transparent documentation of analytic decisions, and transferability was addressed by providing detailed descriptions of the study context, participants, and healthcare setting.

Although the thematic analysis was conducted independently of any predefined framework, the CFIR was later applied as a conceptual lens to guide interpretation. The mapping of inductively generated themes onto CFIR constructs was conducted iteratively and reviewed collaboratively by the research team to ensure conceptual alignment and validity. A detailed mapping of themes and subthemes to the CFIR domains is provided in Table S4 in S1 File.

CFIR offers a comprehensive model for examining multi-level factors influencing healthcare implementation and integrates constructs from more than 20 theoretical models. It was selected for its established utility in evaluating PGx adoption across diverse clinical settings. A recent scoping review of pharmacogenetic studies further supports CFIR’s relevance in identifying common barriers and facilitators to PGx integration, underscoring its suitability for contextualising the present findings within the broader implementation literature [28].

### Ethical considerations

This study was conducted with full ethical approval from the UAEU Social Sciences Ethics Committee on 24/06/2024 (Application No: ERSC_2024_4782), as well as additional approval from the hospital’s ethics committee (Approval No: MF2058-2024-1138). Ensuring participants’ rights, privacy, and comfort was a top priority throughout the research process. Participants received a clear explanation of the study’s purpose and procedures, and informed digital consent was obtained. Participation was voluntary, with the option to withdraw at any time without consequences. All interviews were anonymised, and recordings were securely stored on a password-protected system accessible only to the research team. Transcripts were reviewed to remove any identifying information before analysis. The study adhered to the Declaration of Helsinki and followed best practices for qualitative research, ensuring respect, autonomy, and confidentiality throughout the process.

## Results

### Demographic and professional characteristics of participants

A total of fifteen cardiologists participated in the study. The majority were male (n = 10) and aged 45 years or older (n = 10). Most participants were employed in government healthcare institutions (n = 9), while the remainder worked in the private sector (n = 6). Nine cardiologists had 15 or more years of clinical experience, while six had fewer than 15. In terms of specialisation, the sample included six interventional cardiologists and six non-invasive cardiologists. None of the participants had received formal education or training in PGx (Table S3 in S1 File).

### Thematic analysis

Thematic analysis of interview data identified four overarching themes reflecting cardiologists’ perspectives on the implementation of PGx in cardiovascular care. These themes encompass both systemic and individual dimensions influencing PGx awareness, perceived utility, and readiness for adoption. Subthemes within each category provide finer-grained insight into educational, organisational, and ethical contexts that shape clinical engagement with PGx.

**Fig 1:**
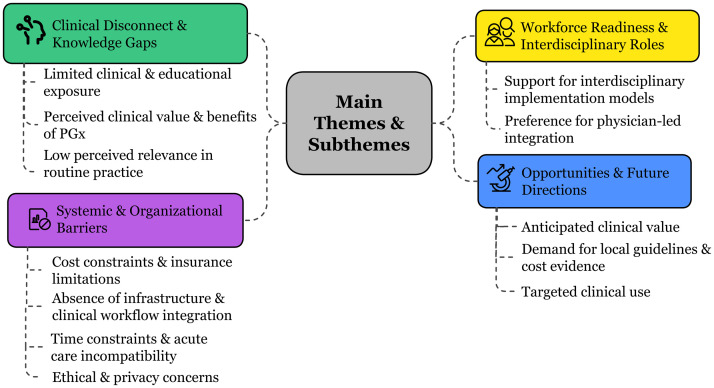
Cardiologists’ perspectives on pharmacogenomics implementation. Thematic map showing major themes and subthemes identified from interviews with cardiologists regarding pharmacogenomics implementation, including knowledge gaps, organizational barriers, workforce readiness, and future opportunities.

Themes were generated inductively through iterative coding and constant comparison. They capture a spectrum of viewpoints ranging from limited awareness and perceived irrelevance to cautious optimism about PGx’s future role in precision cardiology. While some themes are conceptually related, they are presented separately to reflect different levels of influence on PGx implementation, including individual, organisational, and system-level factors. A summary of themes, subthemes, and supporting participant quotations is presented in Table 1, and the conceptual relationships are illustrated in Fig 1. This structure ensures analytic transparency while preserving the diversity of participant experiences and interpretations.

**Table 1:** Summary of themes, subthemes, and representative quotations.

Theme	Subtheme	Summary of Key Findings	Illustrative Quotations
**1. Clinical Disconnect and Knowledge Gaps**	1.1 Limited Clinical and Educational Exposure	Cardiologists had minimal structured exposure to PGx; knowledge was acquired informally through journals or research rather than curricula or CME. PGx was absent from medical school, residency, and fellowship training, and participants emphasised the need for practical, case-based education opportunities.	“I was not really aware of what this is all about… During my training, I did not encounter any such kind of testing.” (M.SH) “I have never been invited to a conference about PGx… there is no dedicated session for it.” (V.P)
	1.2 Low Perceived Relevance in Routine Practice	PGx was viewed as conceptually valuable but of low immediate relevance to routine cardiology. Participants saw it as a future innovation rather than a current necessity and noted that existing fallback strategies made testing seem redundant.	“If I can remember only one patient in 10 years, it does not justify checking every patient for clopidogrel sensitivity.” (M.R) “We don’t really look into statins… if it doesn’t work, we switch to injectables.” (A.M)
	1.3 Perceived Clinical Value and Benefits	Despite limited use, participants recognised PGx’s potential to enhance medication safety, reduce complications, and improve targeting if educational and system barriers were addressed.	“It can avoid complications post-cardiac procedures… simply by only doing this testing.” (S.P) “If we do the tests, we avoid reactions… Reactions cause complications, and complications cost money.” (V.P)
**2. Systemic and Organisational Barriers**	2.1 Cost Constraints and Insurance Limitations	Financial and reimbursement issues were the most frequently cited barriers. PGx testing was perceived as costly, rarely covered by insurance, and inaccessible without health-authority mandates. Clinicians called for preventive reimbursement models recognising long-term savings.	“Insurance companies are unlikely to cover PGx unless it is proven to reduce costs.” (M.SH) “Investing more upfront in preventive measures can reduce hospital admissions and long-term costs.” (A.A)
	2.2 Absence of Infrastructure and Workflow Integration	Participants reported a lack of institutional pathways, limited awareness of testing labs, no EHR integration, and poor dissemination of official guidelines. Information overload and lack of specialised support staff further hindered adoption.	“We do not know which labs provide PGx tests, and we do not have a clear process for ordering them.” (N.T) “Unless someone puts it on my desk, I probably won’t open it.” (G.J)
	2.3 Time Constraints and Acute Care Incompatibility	Turnaround times were incompatible with acute cardiology settings requiring rapid decisions. Participants suggested embedding PGx into preventive care or adopting point-of-care testing for timely results.	“If I need a PGx test result urgently, I cannot afford to wait weeks.” (S.P) “On-the-spot results would be fantastic.” (H.T)
	2.4 Ethical and Privacy Concerns	Concerns centred on patient hesitancy, data privacy, and potential insurance discrimination. Participants linked these issues to the absence of clear data governance frameworks but anticipated improvement through national health databases.	“Some patients do not want their genetic information recorded… they think it will affect insurance.” (M.R) “Genetics is a red flag for insurance. Once they see that word, they close the file.” (M.SH)
**3. Workforce Readiness and Interdisciplinary Roles**	3.1 Support for Interdisciplinary Implementation Models	Many endorsed team-based approaches involving genetic counsellors and clinical pharmacists to interpret results, educate patients, and manage medico-legal complexities.	“We need teamwork on this… A genetic counsellor can help patients understand their results.” (V.M) “There should always be a genetic counsellor… there is also a medical-legal part of it.” (T.SH)
	3.2 Preference for Physician-Led Integration	Others preferred a streamlined physician-led model where PGx is fully embedded into standard workflows with automated CDS.	“Ideally, this should be integrated into the system like any other lab test.” (G.J)
**4. Opportunities and Future Directions for PGx Integration**	4.1 Anticipated Clinical Value and Future Integration	Participants expected PGx to become part of mainstream cardiology once access and cost barriers are addressed, particularly for high-impact drugs like statins, clopidogrel, and warfarin.	“For statins, Plavix, and Warfarin, there is enough evidence. These could be implemented within 2 to 3 years.” (V.M)
	4.2 Demand for Local Guidelines and Cost Evidence	Strong calls were made for UAE-specific clinical guidance and local cost-effectiveness evidence to justify implementation.	“Before making it routine, we need to see if PGx really improves outcomes here.” (T.SH) “There needs to be country-specific guidance to account for our population and system.” (A.M)
	4.3 Selective and Condition-Specific Use of PGx	PGx was seen as most valuable when applied selectively to high-impact or resistant cases rather than universally, balancing benefit with economic feasibility.	“It is not something I would use for every patient.” (M.H)

## Discussion

This study explored cardiologists’ perspectives on the real-world implementation of PGx in the UAE, revealing a complex interplay of systemic, institutional, and individual-level barriers. While PGx was widely regarded as a promising precision tool to improve pharmacotherapy in cardiovascular care, participants highlighted multiple factors that constrained its adoption, including insufficient infrastructure, limited clinical confidence, policy gaps, and a lack of context-specific implementation strategies. These challenges were deeply embedded in the dual-sector structure of the UAE healthcare system and reflected both globally recognised patterns and locally specific dynamics.

To interpret these findings within a structured implementation science lens, the CFIR was applied post hoc. Rather than informing the initial coding, CFIR was used to organise the inductively derived themes across five domains: intervention characteristics, outer setting, inner setting, characteristics of individuals, and implementation process. This abductive approach, which has been adopted in other genomic medicine and implementation studies, enables deeper analytical interpretation while preserving the integrity of emergent insights [27,28,36,37].

**Fig 2:**
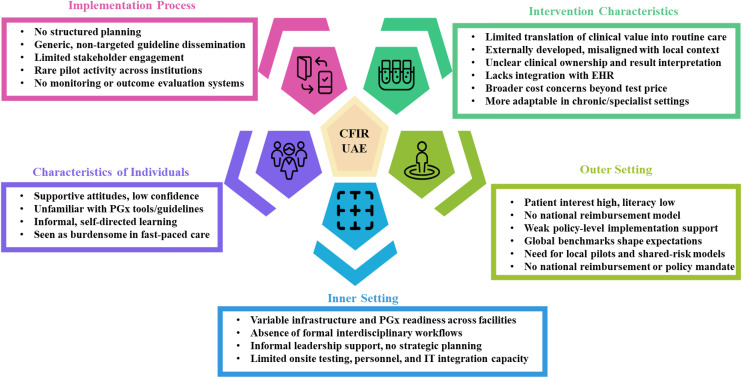
CFIR-guided analysis of pharmacogenomics implementation in UAE cardiology. Mapping of interview findings to the Consolidated Framework for Implementation Research (CFIR) domains, including intervention characteristics, outer setting, inner setting, characteristics of individuals, and implementation process.

The sections that follow are organised by CFIR domain and integrate participant perspectives with relevant literature to explore barriers, facilitators, and strategic opportunities for PGx adoption in UAE cardiology settings (Fig 2).

### Intervention characteristics

This domain captures attributes of the innovation that shape uptake (relative advantage, evidence strength/design quality, complexity, adaptability/compatibility, cost, and trialability) [38].

Relative advantage was widely recognised, with cardiologists noting PGx’s potential to reduce adverse drug reactions and optimise cardiovascular prescribing. However, this perceived benefit has not yet translated into routine clinical use. Participants attributed this gap primarily to the lack of locally relevant outcome data and limited integration into clinical practice, which appeared to weaken confidence and delay adoption. Similar evidence–practice gaps have been reported in high-income settings, where validated gene–drug associations alone have been insufficient to drive implementation without context-specific data [39]

The strength and quality of the evidence also shaped perceptions. PGx was often viewed as originating in high-resource, external settings and as insufficiently tailored to local health system realities, thereby weakening its legitimacy and relevance [40]. Uncertainty around clinical ownership further contributed to implementation delays, reinforcing evidence that unclear roles can hinder adoption of complex interventions [41].

Complexity was a key barrier, particularly in acute cardiology settings requiring rapid decision-making. PGx was seen as burdensome in time-sensitive contexts, largely due to long turnaround times and limited integration into clinical systems. This reflects similar challenges reported in settings lacking real-time informatics support [42]. While participants recognised the value of integrating PGx with EHRs and clinical decision support (CDS), many considered the UAE’s current digital infrastructure underdeveloped for this purpose. Although national platforms such as NABIDH and MALAFFI have advanced digital health capacity, participants noted that these systems do not yet include PGx alerts or CDS, limiting their utility in real-world prescribing [43–45].

Adaptability was discussed in relation to care settings. Several cardiologists suggested that PGx would be more viable in non-acute environments, such as heart failure clinics or outpatient preventive cardiology, where continuity of care allows time for interpretation and patient engagement. This perspective mirrors NHS pilot experiences and highlights a UAE-specific opportunity for phased implementation in specialist settings [46].

Compatibility with existing workflows was viewed as limited. While PGx aligned conceptually with precision medicine, its fit within UAE cardiology was constrained by the pace of acute care, lack of integrated reporting, and reimbursement gaps. Similar barriers have been reported internationally, where poor Electronic Health Record (EHR) integration and complex report formats hinder real-time use [47,48]. In the Middle East, automation challenges in delivering PGx results through EHR systems have further reduced operational compatibility [49].

Cost emerged as a major perceived barrier, extending beyond the test price to include longer consultations, multidisciplinary coordination, and insurer reluctance to reimburse. Without demonstrable reductions in hospitalisations, adverse events, or prescribing inefficiencies, PGx was considered financially unattractive to both clinicians and payers. This uncertainty mirrors global trends, in which even well-validated tools stall without strong economic modelling and sustainable reimbursement frameworks [27,28]. Experiences from the UK and Canada illustrate this challenge: PGx initiatives were delayed or suspended despite strong clinical evidence due to low clinician awareness, fragmented funding, and the absence of sustainable financing models [39]. Some cardiologists drew parallels with telemedicine in the UAE, which initially struggled without alignment with reimbursement [50].

Trialability, the opportunity to pilot PGx before full-scale adoption, was viewed positively by several participants, who suggested small-scale rollouts in selected cardiology clinics to build local evidence, assess workflow impact, and refine integration strategies before broader implementation.

### Outer setting

The CFIR domain of Outer Setting encompasses external factors that influence implementation, including patient needs and resources, external policies and incentives, peer pressure, external partnerships and global engagement [36].

Patient needs and resources emerged as a central influence on perceived PGx readiness. Several participants noted growing interest among younger, digitally engaged patients, who occasionally inquired about genetic factors affecting medication response. However, this interest was not matched by sufficient genomic literacy or awareness of PGx applications, limiting its translation into clinical demand [51–54]. Recent UAE evidence echoes these findings, showing that patients value medication safety and are generally open to proactive PGx testing; however, limited genomic literacy, concerns about cost, and privacy apprehensions remain key barriers to generating sustained clinical demand [55]. This suggests that patient interest alone is insufficient to drive implementation without parallel efforts to improve genomic literacy and access.

External policies and incentives were viewed as crucial for the adoption of PGx. Cardiologists agreed that national endorsement, insurance coverage, and formal support from the Ministry of Health were essential for integration [56]. International examples reinforced this: NHS England’s Genomic Medicine Service and Genome Canada’s national strategies both enabled PGx uptake through funding and policy alignment [57,58].

Engagement with external peers and global benchmarking also shaped attitudes. Many participants cited PGx integration in the U.S., UK, and Canada as hallmarks of modernised healthcare, perceiving adoption in the UAE as an opportunity to enhance institutional reputation and future readiness [59,60]. However, this aspirational view was often tempered by recognition that the UAE is at an earlier stage of implementation, with infrastructure and policy frameworks still evolving.

In the absence of clear national mandates, several cardiologists advocated for locally driven pilot programs as pragmatic entry points. Suggested approaches included public–private partnerships, shared-risk funding, and hospital-level initiatives to generate local outcome data, refine workflows, and build institutional momentum before large-scale adoption. This mirrors successful implementation efforts elsewhere, where pilot studies engaged stakeholders, secured buy-in, and informed scale-up strategies [61]. In the UAE context, locally led pilot initiatives could serve as a practical mechanism for bridging the current policy–practice gap.

### Inner setting

The CFIR domain of Inner Setting refers to the structural, cultural, and operational features within organisations that influence implementation, including structural characteristics, leadership engagement, communication networks, and resource availability [36].

Structural characteristics and infrastructure varied widely between institutions. Cardiology services in the UAE are characterised by differences in infrastructure, decision-making culture, and access to genomic technologies across public and private sectors [31]. Clinicians in tertiary hospitals reported better access to diagnostics, supportive leadership, and electronic systems that support PGx integration, whereas those in smaller or mixed-practice settings described limited capacity and resources. This aligns with global evidence that implementation readiness often depends on facility size, infrastructure, and internal capabilities [58,62].

Networks and communication also influenced readiness. While cardiologists acknowledged the importance of interdisciplinary input, particularly from pharmacists and laboratory professionals, they noted the absence of formal structures to support shared decision-making. This mirrors broader patterns where fragmented communication has hindered PGx uptake and underutilised pharmacist expertise [42].

Leadership engagement emerged as a potential enabler, especially in institutions where department heads encouraged education and exploration. However, this support was often informal and verbal rather than embedded in strategic plans, guidelines, or policy. Such passive endorsement, without formalisation, is a common barrier in implementation, often resulting in stalled or fragmented adoption [63].

Available resources were identified as one of the most immediate barriers. Cardiologists cited the absence of on-site PGx testing facilities, reliance on external laboratories with long turnaround times, and a shortage of trained personnel to support test interpretation and clinical integration. These constraints slowed decision-making and discouraged uptake in fast-paced cardiology settings. Similar challenges are documented in genomic medicine literature, particularly in systems undergoing digital transition or operating in hybrid public–private models [64,65].

In addition, the absence of integrated decision-support tools and genomic informatics capacity may further undermine clinician confidence, as reliance on fragmented information and self-interpretation has been shown to impede safe PGx use [66].

The lack of institutional investment in PGx-specific infrastructure was interpreted not only as a logistical limitation but also as a signal that PGx was not yet prioritised within organisational missions. This reflects findings from global studies where insufficient institutional alignment and resourcing slowed the integration of precision medicine into routine care [67,68].

### Characteristics of individuals

The CFIR domain of Characteristics of Individuals addresses how personal attributes such as knowledge, attitudes, confidence, and readiness for change influence clinicians’ engagement with a new intervention [28]. Cardiologists expressed general support for PGx, particularly its potential to improve drug safety, but this was not matched by practical readiness

Many participants reported low confidence in ordering tests, interpreting results, and integrating findings into treatment decisions, especially under time constraints.This highlights a gap between positive attitudes and self-efficacy, a pattern also observed in other healthcare settings [34].

Limited exposure to formal PGx education further compounded low confidence. Most cardiologists were unfamiliar with internationally endorsed implementation resources [11,69]. None of the participants recalled PGx-focused lectures, workshops, or CME events during training or practice. In the absence of structured educational programs or institutional protocols, learning was inconsistent, self-directed, and often reliant on informal reading or rare clinical cases. This mirrors findings from other regions, where low familiarity with PGx resources has been identified as a core barrier to adoption [70,71], reinforcing the need for targeted professional development and integration of PGx into cardiology-specific training pathways [72].

Several cardiologists perceived PGx as burdensome rather than facilitative. In fast-paced environments, unclear responsibilities, logistical complexity, and lack of decision-support tools were cited as deterrents. These perceptions suggest that when PGx is seen as adding workload or disrupting workflow, adoption is unlikely despite favourable attitudes, consistent with broader implementation literature [12,73].

### Implementation process

The CFIR domain of Implementation Process captures the planning, engagement, execution, and evaluation activities that drive an innovation from concept to routine use [38]. In this study, cardiologists identified multiple gaps in process design, stakeholder coordination, and institutional follow-through, reflecting a healthcare environment where PGx is supported in theory but remains underdeveloped in practice.

National PGx guidelines exist in the UAE, yet participants reported low awareness and limited uptake in cardiology. They noted that existing guidance was often shared through generic circulars lacking adaptation to cardiovascular workflows or time-pressured decision-making. This reflects global concerns that national strategies without frontline tailoring rarely achieve effective implementation [74].

Stakeholder coordination was described as minimal, with disjointed communication between laboratories, insurers, and allied health professionals and no formal multidisciplinary workflows. International examples, including the IGNITE Network and NHS Genomic Medicine Service, highlight that structured engagement through task forces and advisory committees can be critical for system-wide PGx adoption [74–76]

Only one participant reported involvement in a PGx-related study, and others noted no local pilot projects to establish feasibility. Globally, pilot initiatives have been shown to demonstrate operational viability, generate context-specific outcome data, and secure institutional and payer support [74].

Participants also reported no evaluation mechanisms, including systems to monitor test use, prescribing changes, or patient outcomes. Without such feedback systems, sustained implementation is unlikely, as monitoring and evaluation are critical components of successful integration [73].

Beyond its practical implications, this study contributes to implementation science by applying the CFIR within a hybrid public–private healthcare system and a cardiology-specific context. The findings highlight how implementation factors interact across multiple levels, particularly in settings characterised by fragmented workflows, variable resources, and evolving policy environments. This reinforces the value of CFIR as a flexible framework for understanding context-dependent implementation challenges and extends its application to pharmacogenomics in cardiology within hybrid health systems.

**Fig 3:**
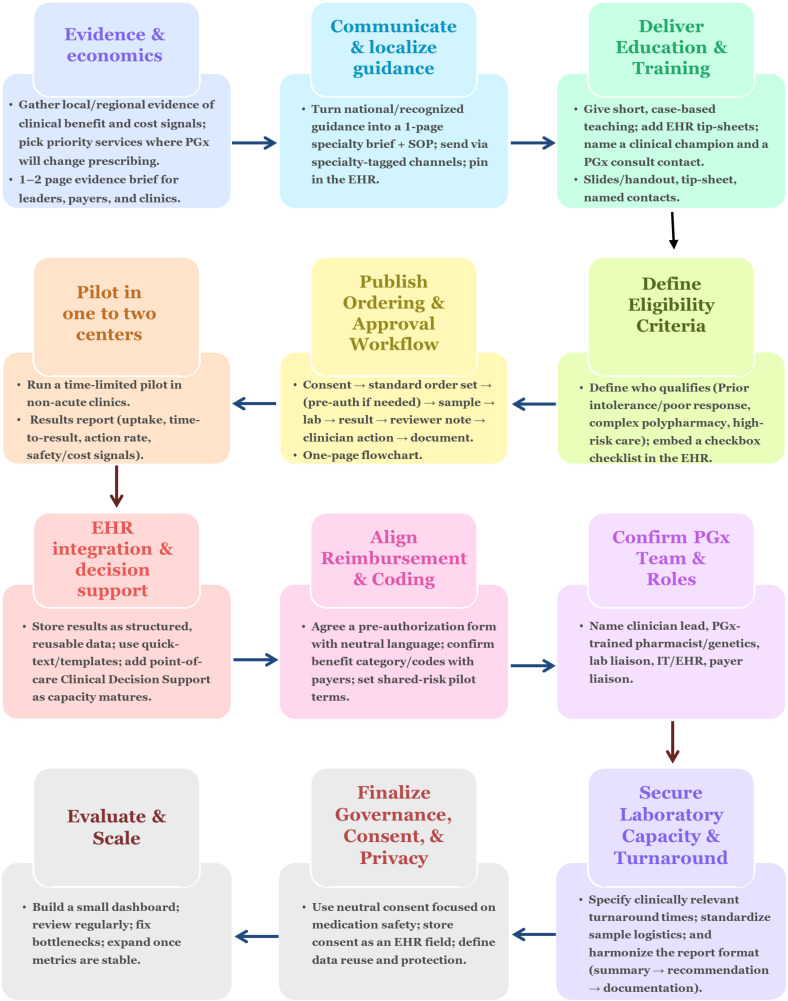
Proposed implementation framework for pharmacogenomics in hybrid health systems. Stepwise framework outlining key actions for pharmacogenomics implementation, including evidence generation, guideline localization, workforce training, workflow integration, reimbursement alignment, governance, and evaluation.

Building on these findings, a stepwise implementation framework is proposed to support the integration of PGx across services. The framework comprises twelve linked steps: evidence synthesis; tailored communication of guidance; short case-based training; clear eligibility criteria; a one-page ordering/approval workflow; a time-limited pilot in one to two centres; EHR integration (structured results with point-of-care support); reimbursement and coding alignment; role clarity (including a PGx-trained pharmacist or genetics clinician); laboratory capacity with timely turnaround and standardised reporting; governance, consent, and privacy; and dashboard-led evaluation with phased scale-up (Fig 3).

### Strengths and limitations

This study provides early, practice-grounded insights into the implementation of PGx in a hybrid health system, drawing on accounts from practising cardiologists across public and private hospitals. The use of the CFIR enabled a structured, multilevel analysis and facilitated comparison with international literature.

Some limitations should be considered. First, the study focused only on cardiologists and did not include other key stakeholders such as patients, pharmacists, payers, or laboratory professionals, which may limit the breadth of perspectives captured. Second, the use of purposive, convenience, and snowball sampling may introduce selection bias, as participants may have been more familiar with or positively inclined toward PGx, potentially influencing the views reported and limiting generalisability. Third, as the study was conducted in a single country, the findings may not be fully transferable to other healthcare systems.

In addition, most participants had limited formal education or hands-on experience in PGx, which may have shaped their perceptions and affected the transferability of findings to settings with more established PGx implementation. Although online interviews enabled participation across multiple regions, this format may have limited the ability to capture nonverbal cues and influenced rapport during data collection. Future research should adopt multi-stakeholder and mixed-method approaches to triangulate findings and support more scalable implementation strategies.

## Conclusion

In a hybrid public–private context, cardiologists viewed PGx as clinically valuable but constrained by workflow, infrastructure, reimbursement, and gaps in role clarity. Using the CFIR, this study translates those determinants into an implementation framework with concrete steps, including targeted guidance, education, piloting in non-acute pathways, structured EHR integration, payer alignment, team-based processes, and turnaround standards. These actions provide a practical route to responsible, scalable, and equity-minded PGx adoption in cardiology and other clinical departments.

## Supporting information

S1 File Supplementary materials. This file contains: (1) the COREQ checklist (Table S1), (2) the semi-structured interview guide (Table S2), (3) participant demographic and professional characteristics (Table S3), and (4) mapping of themes and subthemes to CFIR domains (Table S4).
(DOCX)

## Abbreviations

PGx: Pharmacogenomics
CFIR: Consolidated Framework for Implementation Research
UAE: United Arab Emirates
CME: Continuing Medical Education
CPIC: Clinical Pharmacogenetics Implementation Consortium
DPWG: Dutch Pharmacogenetics Working Group
FDA: U.S. Food and Drug Administration
EMA: European Medicines Agency
EHR: Electronic Health Record
DOH: Department of Health (Abu Dhabi)
Nvivo: Qualitative Data Analysis Software
COREQ: Consolidated Criteria for Reporting Qualitative Research
CVD: Cardiovascular Disease

## References

[1] Roden DM, Wilke RA, Kroemer HK, Stein CM. Pharmacogenomics: the genetics of variable drug responses. Circulation. 2011;123(15):1661–70. doi: 10.1161/CIRCULATIONAHA.109.914820 21502584 PMC3093198

[2] T P A, M SS, Jose A, Chandran L, Zachariah SM. Pharmacogenomics: the right drug to the right person. J Clin Med Res. 2009;1(4):191–4. doi: 10.4021/jocmr2009.08.1255 22461867 PMC3299179

[3] Duarte JD, Cavallari LH. Pharmacogenetics to guide cardiovascular drug therapy. Nat Rev Cardiol. 2021;18(9):649–65. doi: 10.1038/s41569-021-00549-w 33953382 PMC8364496

[4] Zhou Z. Pharmacogenomics in cardiovascular precision medicine. J Lab Precis Med. 2020;5:30. doi: 10.21037/JLPM-2019-CPM-05

[5] Kee PS, Chin PKL, Kennedy MA, Maggo SDS. Pharmacogenetics of statin-induced myotoxicity. Front Genet. 2020;11:575678. doi: 10.3389/fgene.2020.575678 33193687 PMC7596698

[6] Bozina N. The pharmacogenetics of warfarin in clinical practice. Biochem Med (Zagreb). 2010;20(1):33–44. doi: 10.11613/BM.2010.005

[7] Lopez J, Mark J, Duarte GJ, Shaban M, Sosa F, Mishra R, et al. Role of genetic polymorphisms in clopidogrel response variability: a systematic review. Open Heart. 2023;10(2):e002436. doi: 10.1136/openhrt-2023-002436 37963685 PMC10649851

[8] CPIC. Guidelines [Internet]. [cited 2024 Nov 10]. Available from: https://cpicpgx.org/guidelines/

[9] Committee for Medicinal Products for Human Use (CHMP). Guideline on the use of pharmacogenetic methodologies in the pharmacokinetic evaluation of medicinal products [Internet]. 2009 [cited 2025 Apr 2]. Available from: https://www.ema.europa.eu/

[10] US Food and Drug Administration. Table of pharmacogenomic biomarkers in drug labeling [Internet]. 2022 [cited 2025 Apr 2]. Available from: https://www.fda.gov/drugs/science-and-research-drugs/table-pharmacogenomic-biomarkers-drug-labeling

[11] Dutch Pharmacogenetics Working Group. DPWG: Dutch Pharmacogenetics Working Group [Internet]. 2025 [cited 2025 Apr 2]. Available from: https://www.pharmgkb.org/page/dpwg

[12] Wu J. Integrating pharmacogenomics into treatments: rationales, current challenges, and future directions. Georgetown Med Rev. 2022;6(1). doi: 10.52504/001c.37021

[13] Haga SB, Burke W. Using pharmacogenetics to improve drug safety and efficacy. JAMA. 2004;291(23):2869–71. doi: 10.1001/jama.291.23.2869 15199039

[14] Wong WB, Carlson JJ, Thariani R, Veenstra DL. Cost effectiveness of pharmacogenomics: a critical and systematic review. Pharmacoeconomics. 2010;28(11):1001–13. doi: 10.2165/11537410-000000000-00000 20936884

[15] Cohen J, Wilson A, Manzolillo K. Clinical and economic challenges facing pharmacogenomics. Pharmacogenomics J. 2013;13(4):378–88. doi: 10.1038/tpj.2011.63 22231566

[16] Ateia H, Ogrodzki P, Wilson HV, Ganesan S, Halwani R, Koshy A, et al. Population genome programs across the Middle East and North Africa: successes, challenges, and future directions. Biomed Hub. 2023;8(1):60–71. doi: 10.1159/000530619 37900972 PMC10601860

[17] Rahma AT, Elbarazi I, Ali BR, Patrinos GP, Ahmed LA, Al Maskari F. Genomics and pharmacogenomics knowledge, attitude and practice of pharmacists working in United Arab Emirates: findings from focus group discussions-a qualitative study. J Pers Med. 2020;10(3):134. doi: 10.3390/jpm10030134 32962013 PMC7563679

[18] Rahma AT, Elsheik M, Ali BR, Elbarazi I, Patrinos GP, Ahmed LA, et al. Knowledge, attitudes, and perceived barriers toward genetic testing and pharmacogenomics among healthcare workers in the United Arab Emirates: a cross-sectional study. J Pers Med. 2020;10(4):216. doi: 10.3390/jpm10040216 33182317 PMC7711841

[19] Khattab M, Baguneid M, Ali BR, Sadek B, Beiram R, Atallah B. A review of pharmacogenomics studies assessing the knowledge and attitudes of physicians and pharmacists across the Arab and Middle Eastern region. Pharm Pract (Granada). 2023;21(3):2828. doi: 10.18549/PharmPract.2023.3.2828

[20] Jairoun AA, Al-Hemyari SS, Shahwan M, Al-Ani M, Yaseen MA, Al-Aawad MH, et al. Empowering precision medicine: Insights from a national survey on pharmacogenomics knowledge, attitudes, and perceptions among community pharmacists in the UAE. Explor Res Clin Soc Pharm. 2024;16:100508. doi: 10.1016/j.rcsop.2024.100508 39376795 PMC11456781

[21] Alqasrawi MN, Al-Mahayri ZN, Alblooshi H, Alsafar H, Ali BR. Utilizing pharmacogenomic data for a safer use of statins among the Emirati population. Curr Vasc Pharmacol. 2024;22(3):218–29. doi: 10.2174/0115701611283841231227064343 38284696

[22] Alqasrawi MN, Al-Mahayri ZN, AlBawa’neh AS, Khasawneh LQ, Dabaghie L, Altoum SM, et al. Pharmacogenomic insights into atorvastatin and rosuvastatin adverse effects: a prospective observational study in the UAE’s multiethnic population. Hum Genomics. 2025;19(1):44. doi: 10.1186/s40246-025-00753-6 40281622 PMC12032684

[23] Khasawneh LQ, Alsafar H, Alblooshi H, Allam M, Patrinos GP, Ali BR. The diversity and clinical implications of genetic variants influencing clopidogrel bioactivation and response in the Emirati population. Hum Genomics. 2024;18(1):2. doi: 10.1186/s40246-023-00568-3 38173046 PMC10765826

[24] Al-Mahayri ZN, Khasawneh LQ, Alqasrawi MN, Altoum SM, Jamil G, Badawi S, et al. Pharmacogenomics implementation in cardiovascular disease in a highly diverse population: initial findings and lessons learned from a pilot study in United Arab Emirates. Hum Genomics. 2022;16(1):42. doi: 10.1186/s40246-022-00417-9 36154845 PMC9509637

[25] Department of Health Abu Dhabi. Pharmacogenomics (PGx) guideline [Internet]. Version V1. Document Ref. No.: DOH/GD/RIC/PG/V1/2024. Abu Dhabi: Department of Health Abu Dhabi, Research and Innovation Center, Genome Division; 2024.

[26] Husereau D, Drummond M, Augustovski F, de Bekker-Grob E, Briggs AH, Carswell C, et al. Consolidated health economic evaluation reporting standards 2022 (CHEERS 2022) statement: updated reporting guidance for health economic evaluations. MDM Policy Pract. 2022;7(1). doi: 10.1177/23814683211061097 PMC8755935 35036563

[27] McDermott JH, Wright S, Sharma V, Newman WG, Payne K, Wilson P. Characterizing pharmacogenetic programs using the consolidated framework for implementation research: a structured scoping review. Front Med (Lausanne). 2022;9:945352. doi: 10.3389/fmed.2022.945352 36059837 PMC9433561

[28] Damschroder LJ, Aron DC, Keith RE, Kirsh SR, Alexander JA, Lowery JC. Fostering implementation of health services research findings into practice: a consolidated framework for advancing implementation science. Implement Sci. 2009;4:50. doi: 10.1186/1748-5908-4-50 19664226 PMC2736161

[29] Tong A, Sainsbury P, Craig J. Consolidated criteria for reporting qualitative research (COREQ): a 32-item checklist for interviews and focus groups. Int J Qual Health Care. 2007;19(6):349–57. doi: 10.1093/intqhc/mzm042 17872937

[30] Koornneef E, Robben P, Blair I. Progress and outcomes of health systems reform in the United Arab Emirates: a systematic review. BMC Health Serv Res. 2017;17(1):672. doi: 10.1186/s12913-017-2597-1 28931388 PMC5607589

[31] Blair I, Sharif A. Health and health systems performance in the United Arab Emirates. World Hosp Health Serv. 2013;49(4):12–7. 24683809

[32] Rigter T, Jansen ME, de Groot JM, Janssen SWJ, Rodenburg W, Cornel MC. Implementation of pharmacogenetics in primary care: a multi-stakeholder perspective. Front Genet. 2020;11:10. doi: 10.3389/fgene.2020.00010 32076434 PMC7006602

[33] Romagnoli KM, Boyce RD, Empey PE, Adams S, Hochheiser H. Bringing clinical pharmacogenomics information to pharmacists: a qualitative study of information needs and resource requirements. Int J Med Inform. 2016;86:54–61. doi: 10.1016/j.ijmedinf.2015.11.015 26725696 PMC4720137

[34] Koufaki M-I, Patrinos GP, Vasileiou KZ. A qualitative approach to assess the opinion of physicians about the challenges and prospects of pharmacogenomic testing implementation in clinical practice in Greece. Hum Genomics. 2024;18(1):82. doi: 10.1186/s40246-024-00648-y 39030587 PMC11264745

[35] Saunders B, Sim J, Kingstone T, Baker S, Waterfield J, Bartlam B, et al. Saturation in qualitative research: exploring its conceptualization and operationalization. Qual Quant. 2018;52(4):1893–907. doi: 10.1007/s11135-017-0574-8 29937585 PMC5993836

[36] Smith LR, Damschroder L, Lewis CC, Weiner B. The consolidated framework for implementation research: advancing implementation science through real-world applications, adaptations, and measurement. Implement Sci. 2015;10(Suppl 1):A11. doi: 10.1186/1748-5908-10-S1-A11

[37] Orlando LA, Sperber NR, Voils C, Nichols M, Myers RA, Wu RR, et al. Developing a common framework for evaluating the implementation of genomic medicine interventions in clinical care: the IGNITE Network’s Common Measures Working Group. Genet Med. 2018;20(6):655–63. doi: 10.1038/gim.2017.144 28914267 PMC5851794

[38] Damschroder L, Hall C, Gillon L, Reardon C, Kelley C, Sparks J. The Consolidated Framework for Implementation Research (CFIR): progress to date, tools and resources, and plans for the future. Implement Sci. 2015;10(Suppl 1):A12. doi: 10.1186/1748-5908-10-S1-A12

[39] Cooper J, Pratt J, Park J, Fahim C, Lovnicki JM, Groeneweg GSS, et al. Implementation of pharmacogenetic testing in pediatric oncology: barriers and facilitators assessment at eight Canadian academic health centres. Pharmacogenomics J. 2024;24(6):36. doi: 10.1038/s41397-024-00356-9 39562543

[40] Vest BM, Wray LO, Brady LA, Thase ME, Beehler GP, Chapman SR, et al. Primary care and mental health providers’ perceptions of implementation of pharmacogenetics testing for depression prescribing. BMC Psychiatry. 2020;20(1):518. doi: 10.1186/s12888-020-02919-z 33115428 PMC7594429

[41] Bernards B. Cognitive uncertainty and employees’ daily innovative work behavior: the moderating role of ambidextrous leadership. Rev Public Personnel Admin. 2024;45(4):647–70. doi: 10.1177/0734371x241233759

[42] Bosic-Reiniger J, Martin JL, Brown KE, Anderson HD, Blackburn H, Kao DP, et al. Barriers and facilitators of the use of clinical informatics resources to facilitate pharmacogenomic implementation in resource-limited settings. JAMIA Open. 2024;7(4):ooae101. doi: 10.1093/jamiaopen/ooae101 39399271 PMC11471000

[43] McDermott JH, Sharma V, Keen J, Newman WG. Embedding pharmacogenetics into clinical practice to improve patient outcomes. Ann Hum Genet. 2025;89(5):398–405. doi: 10.1111/ahg.12601 40358420 PMC12336961

[44] Malaffi. About Malaffi [Internet]. [cited 2024 Dec 3]. Available from: https://malaffi.ae/

[45] NABIDH [Internet]. 2020 [cited 2024 Dec 3]. Available from: https://nabidh.ae/#/comm/landing

[46] Berth H, Balck F, Dinkel A. Attitudes toward genetic testing in patients at risk for HNPCC/FAP and the German population. Genet Test. 2002;6(4):273–80. doi: 10.1089/10906570260471804 12537651

[47] Rogers EM, Singhal A, Quinlan MM. Diffusion of innovations. In: Salwen MB, Stacks DW, editors. An integrated approach to communication theory and research. New York: Routledge; 2014. pp. 432–48. doi: 10.4324/9780203887011-36

[48] Swen JJ, van der Wouden CH, Manson LE, Abdullah-Koolmees H, Blagec K, Blagus T, et al. A 12-gene pharmacogenetic panel to prevent adverse drug reactions: an open-label, multicentre, controlled, cluster-randomised crossover implementation study. Lancet. 2023;401(10374):347–56. doi: 10.1016/S0140-6736(22)01841-4 36739136

[49] El Shamieh S, Saleem RA, Hammoudi Halat D, Fakhoury HMA, Bastaki K, Fawaz M, et al. Integrating pharmacogenomics in three Middle Eastern countries’ healthcare (Lebanon, Qatar, and Saudi Arabia): Current insights, challenges, and strategic directions. PLoS One. 2025;20(4):e0319042. doi: 10.1371/journal.pone.0319042 40215419 PMC11991729

[50] Hussain KHH, Al Shmanee MZ, Taha FH, Samara KA, Barqawi HJ, Dash NR. Perception, usability, and satisfaction with telemedicine in the United Arab Emirates. Telemed J E Health. 2024. doi: 10.1089/tmj.2024.0207 39072672

[51] Haga SB, Mills R, Moaddeb J, Allen Lapointe N, Cho A, Ginsburg GS. Patient experiences with pharmacogenetic testing in a primary care setting. Pharmacogenomics. 2016;17(15):1629–36. doi: 10.2217/pgs-2016-0077 27648637 PMC5558503

[52] Haga SB, O’Daniel JM, Tindall GM, Lipkus IR, Agans R. Survey of U.S. public attitudes towards pharmacogenetic testing. Pharmacogenomics J. 2012;12(3):197–204. doi: 10.1038/tpj.2011.1 21321582 PMC3139751

[53] Zhang L, Jacobson PA, Johnson ANK, Gregornik DB, Johnson SG, McCarty CA, et al. Public Attitudes toward pharmacogenomic testing and establishing a statewide pharmacogenomics database in the State of Minnesota. J Pers Med. 2022;12(10):1615. doi: 10.3390/jpm12101615 36294754 PMC9604616

[54] Rahma AT, Ali BR, Patrinos GP, Ahmed LA, Elbarazi I, Abdullahi AS, et al. Knowledge, attitudes, and perceptions of the multi-ethnic population of the United Arab Emirates on genomic medicine and genetic testing. Hum Genomics. 2023;17(1):63. doi: 10.1186/s40246-023-00509-0 37454085 PMC10349494

[55] Abbas MO, Rahma AT, Elbarazi I, Ali BR, Patrinos GP, Nagy F. Knowledge and views of patients with cardiovascular disease toward pharmacogenomics in the United Arab Emirates. Clin Transl Sci. 2025;18(8):e70300. doi: 10.1111/cts.70300 PMC12338313 40788819

[56] Abbas MO, Rahma AT, Elbarazi I, Ali BR, Patrinos GP, Ghadibah H, et al. Strategic insights into pharmacogenomics coverage: a theory-informed SWOT analysis of UAE insurance stakeholders’ perspectives. Hum Genomics. 2025;20(1):26. doi: 10.1186/s40246-025-00896-6 41462354 PMC12860024

[57] McDermott J. Determining the clinical utility of panel-based pharmacogenetic testing and defining optimal models for implementation. Manchester (UK): The University of Manchester; 2024.

[58] Friedrich B, Vindrola-Padros C, Lucassen AM, Patch C, Clarke A, Lakhanpaul M, et al. “A very big challenge”: a qualitative study to explore the early barriers and enablers to implementing a national genomic medicine service in England. Front Genet. 2024;14:1282034. doi: 10.3389/fgene.2023.1282034 38239852 PMC10794539

[59] Ontario Health. Pharmacogenomics: recommendations for Ontario. Toronto (ON): Ontario Health; 2023. https://ontariohealth.ca/content/dam/ontariohealth/documents/pharmacogenomics-recommendation.pdf

[60] NHS England. NHS Genomic Medicine Service [Internet]. [cited 2025 Jun 2]. Available from: https://www.england.nhs.uk/genomics/nhs-genomic-med-service/

[61] Hellstrand Tang U, Smith F, Karilampi UL, Gremyr A. Exploring the role of complexity in health care technology bottom-up innovations: multiple-case study using the nonadoption, abandonment, scale-up, spread, and sustainability complexity assessment tool. JMIR Hum Factors. 2024;11:e50889. doi: 10.2196/50889 38669076 PMC11087855

[62] Best S, Stark Z, Brown H, Long JC, Hewage K, Gaff C, et al. The leadership behaviors needed to implement clinical genomics at scale: a qualitative study. Genet Med. 2020;22(8):1384–90. doi: 10.1038/s41436-020-0818-1 32398772 PMC7394877

[63] Landis BJ, Ware SM. The current landscape of genetic testing in cardiovascular malformations: opportunities and challenges. Front Cardiovasc Med. 2016;3:22. doi: 10.3389/fcvm.2016.00022 27504451 PMC4959014

[64] Mai C-W, Sridhar SB, Karattuthodi MS, Ganesan PM, Shareef J, Lee EL, et al. Scoping review of enablers and challenges of implementing pharmacogenomics testing in the primary care settings. BMJ Open. 2024;14(11):e087064. doi: 10.1136/bmjopen-2024-087064 39500605 PMC11552560

[65] Rogers SL. The current state of pharmacoeconomics and reimbursement for pharmacogenomics. Adv Mol Pathol. 2023;6(1):87–97. doi: 10.1016/j.yamp.2023.06.002

[66] Roberts JS, Dolinoy D, Tarini B. Emerging issues in public health genomics. Annu Rev Genomics Hum Genet. 2014;15:461–80. doi: 10.1146/annurev-genom-090413-025514 25184533 PMC4229014

[67] Lemke LK, Alam B, Williams R, Starostik P, Cavallari LH, Cicali EJ, et al. Reimbursement of pharmacogenetic tests at a tertiary academic medical center in the United States. Front Pharmacol. 2023;14:1179364. doi: 10.3389/fphar.2023.1179364 37645439 PMC10461057

[68] van der Wouden CH, Cambon-Thomsen A, Cecchin E, Cheung KC, Dávila-Fajardo CL, Deneer VH, et al. Implementing pharmacogenomics in Europe: design and implementation strategy of the Ubiquitous Pharmacogenomics Consortium. Clin Pharmacol Ther. 2017;101(3):341–58. doi: 10.1002/cpt.602 28027596

[69] Peruzzi E, Roncato R, De Mattia E, Bignucolo A, Swen JJ, Guchelaar H-J, et al. Implementation of pre-emptive testing of a pharmacogenomic panel in clinical practice: Where do we stand? Br J Clin Pharmacol. 2025;91(2):270–82. doi: 10.1111/bcp.15956 37926674 PMC11773130

[70] Caudle KE, Keeling NJ, Klein TE, Whirl-Carrillo M, Pratt VM, Hoffman JM. Standardization can accelerate the adoption of pharmacogenomics: current status and the path forward. Pharmacogenomics. 2018;19(10):847–60. doi: 10.2217/pgs-2018-0028 29914287 PMC6123879

[71] Murad MH, Varkey P. Self-directed learning in health professions education. Ann Acad Med Singap. 2008;37(7):580–90. doi: 10.47102/annals-acadmedsg.v37n7p580 18695772

[72] Goetz LH, Schork NJ. Personalized medicine: motivation, challenges, and progress. Fertil Steril. 2018;109(6):952–63. doi: 10.1016/j.fertnstert.2018.05.006 29935653 PMC6366451

[73] Shugg T, Tillman EM, Breman AM, Hodge JC, McDonald CA, Ly RC, et al. Development of a multifaceted program for pharmacogenetics adoption at an academic medical center: practical considerations and lessons learned. Clin Pharmacol Ther. 2024;116(4):914–31. doi: 10.1002/cpt.3402 39169556 PMC11452286

[74] Wiss FM, Jakober D, Lampert ML, Allemann SS. Overcoming barriers: strategies for implementing pharmacist-led pharmacogenetic services in swiss clinical practice. Genes (Basel). 2024;15(7):862. doi: 10.3390/genes15070862 39062642 PMC11276441

[75] Pires R, Santos MP, Brandão B, Rosa L. Underserving the disadvantaged: institutional failures and their consequences for frontline workers and vulnerable publics. Street-level bureaucracy in weak state institutions. Bristol (UK): Policy Press; 2024. pp. 104–19. doi: 10.51952/9781447368779.ch006

[76] Stewart S, Dodero-Anillo JM, Guijarro-Eguinoa J, Arias P, Gómez López De Las Huertas A, Seco-Meseguer E, et al. Advancing pharmacogenetic testing in a tertiary hospital: a retrospective analysis after 10 years of activity. Front Pharmacol. 2023;14:1292416. doi: 10.3389/fphar.2023.1292416 37927587 PMC10622662

