# Supplementary material for: Cardiologists’ perspectives on pharmacogenomics implementation in a hybrid health system: A qualitative study from the United Arab Emirates
Source: PLoS One. 2026 Jun 12;21(6):e0351218. doi: 10.1371/journal.pone.0351218 (PMC13262839; doi:10.1371/journal.pone.0351218)
Supplement: S1 File — This file contains: (1) the COREQ checklist (Table S1), (2) the semi-structured interview guide (Table S2), (3) participant demographic and professional characteristics (Table S3), and (4) mapping of themes and subthemes to CFIR domains (Table S4). (DOCX) [file pone.0351218.s001.docx]

**Supplementary Materials**

***Table S1: COREQ Checklist***

| Domain | Topic | Item No. | Description | Reported |
| --- | --- | --- | --- | --- |
| Domain 1: Research Team and Reflexivity | Personal Characteristics | 1 | Which author/s conducted the interview or focus group? | Maram O Abbas |
|  |  | 2 | What were the researcher’s credentials? | PhD Candidate |
|  |  | 3 | What was their occupation at the time of the study? | Student |
|  |  | 4 | Was the researcher male or female? | Female |
|  |  | 5 | What experience or training did the researcher have? | Comprehensive training in qualitative methods, including thematic analysis and interview experience. |
|  | Relationship with Participants | 6 | Was a relationship established prior to study commencement? | No |
|  |  | 7 | What did the participants know about the researcher? | Academic background, study objectives. |
|  |  | 8 | What characteristics were reported about the interviewer/facilitator? | Background in public health, qualitative research experience. |
| Domain 2: Study Design | Theoretical Framework | 9 | Methodological orientation stated to underpin the study? | Thematic analysis and CIFR framework |
|  | Participant Selection | 10 | How were participants selected? | Purposive, convenience, and snowballing |
|  |  | 11 | How were participants approached? | Online meetings |
|  |  | 15 | How many participants were in the study? | 15 |
|  |  | 13 | How many people refused to participate or dropped out? Reasons? | 6 (scheduling conflicts) |
|  | Setting | 14 | Where was the data collected? | Virtually via Microsoft Teams |
|  |  | 15 | Was anyone else present besides the participants and researchers? | No |
|  |  | 16 | Important characteristics of the sample? | Practicing cardiologists across different subspecialties, including general cardiology, heart failure, and adult congenital heart disease specialists |
|  | Data Collection | 17 | Were questions/prompts provided? | Yes, developed from literature and expert feedback. |
|  |  | 18 | Were repeat interviews carried out? | No |
|  |  | 19 | Was audio/visual recording used? | Yes |
|  |  | 20 | Were field notes made? | Yes |
|  |  | 21 | Duration of interviews/focus groups? | 30-40 minutes |
|  |  | 22 | Was data saturation discussed? | Yes |
|  |  | 23 | Were transcripts returned to participants for comment and/or correction? | Yes (3 participants) |
| Domain 3: Analysis and Findings | Data Analysis | 24 | Number of data coders? | 1 |
|  |  | 25 | Was a coding tree description provided? | Yes |
|  |  | 26 | Were themes identified in advance or derived from the data? | Derived from data |
|  |  | 27 | What software was used to manage the data? | NVivo |
|  |  | 28 | Did participants provide feedback on the findings? | No |
|  | Reporting | 29 | Were participant quotations presented to illustrate findings? | Yes |
|  |  | 30 | Was there consistency between data and findings? | Yes |
|  |  | 31 | Were major themes clearly presented? | Yes |
|  |  | 32 | Were minor themes described or discussed? | Yes |

***Table S2: Interview Guide***

| Section | Questions |
| --- | --- |
| Demographics | 1. Gender 2. Age 3. Highest Education Level 4. Field of education 5. Name of the institution 6. Years of experience in cardiology 7. Previous positions 8. Current position |
| Awareness and Use of PGx Testing | 1. Have you heard of pharmacogenomic (PGx) testing before? If so, where did you learn about it? |
|  | 1. Have you ever recommended a PGx test for a patient? If yes, in what context? |
|  | 1. Are there specific clinical scenarios or conditions in your specialty where you believe PGx testing is particularly useful? |
|  | 1. How do you perceive the role of PGx testing in your daily clinical practice? |
|  | 1. Have you attended any training or educational sessions about PGx testing? If not, why? |
| Sources of Information | 1. What sources of information do you typically rely on to learn about new medical advancements, such as PGx? |
|  | 1. Do you feel that current sources of PGx information (e.g., journals, guidelines, or conferences) are adequate? |
|  | 1. Have you interacted with any pharmaceutical companies, laboratories, or professional bodies regarding PGx testing? If so, in what capacity? |
| Key Stakeholders and Interactions | 1. Who do you see as the key stakeholders in implementing PGx testing in clinical practice? |
|  | 1. How do you view the interaction between physicians, laboratories, genetic counselors, and other stakeholders in the PGx supply chain? |
|  | 1. What role do you think patients play in driving the adoption of PGx testing? |
|  | 1. Are there any barriers to effective communication or collaboration between stakeholders in PGx implementation? |
|  | 1. Who do you consider as the most influential change agents in advancing PGx adoption (e.g., physicians, insurers, laboratories)? |
| Benefits and Usefulness of PGx Testing | 1. What are the main clinical benefits of PGx testing, in your opinion? |
|  | 1. How does PGx testing contribute to personalized medicine in your specialty? |
|  | 1. Do you think PGx testing can improve patient outcomes, such as reducing adverse drug reactions (ADRs)? |
|  | 1. How do you think PGx testing impacts healthcare costs or resource utilization? |
|  | 1. Are there any specific cases where PGx testing has had a significant impact on patient care in your practice? |
| Barriers to PGx Implementation | 1. What do you see as the primary barriers to implementing PGx testing in clinical practice? |
|  | 1. How does the cost of PGx testing affect its accessibility and adoption? |
|  | 1. Are there any specific challenges related to the reimbursement of PGx testing by insurance providers? |
|  | 1. Do you believe there is sufficient infrastructure and trained personnel to support PGx testing in your region? |
|  | 1. How does the time required for PGx testing results impact its feasibility in urgent clinical settings? |
|  | 1. Are there concerns about patient privacy, ethical issues, or psychological impact related to PGx testing? |
| Recommendations and Future Prospects | 1. What steps do you think could help boost the adoption of PGx testing in clinical practice? |
|  | 1. How can physicians be better trained or informed about PGx testing applications? |
|  | 1. What role do you think public health initiatives or policies should play in promoting PGx testing? |
|  | 1. Do you think reducing the cost or ensuring reimbursement of PGx testing would significantly increase its use? |
|  | 1. How do you envision the role of PGx testing evolving in your specialty over the next 5–10 years? |

Table S3. Demographic and Professional Characteristics of Participating Cardiologists

| Characteristic |  | N (15) |
| --- | --- | --- |
| Gender | Male | 10 |
|  | Female | 5 |
| Age | < 45 years | 5 |
|  | ≥ 45 years | 10 |
| Sector | Government | 9 |
|  | Private | 6 |
| Years of Experience | Less than 15 years | 6 |
|  | 15 years or more | 9 |
| Specialty | Interventional Cardiologists | 6 |
|  | Non-Invasive Cardiologists | 6 |
|  | Cardiothoracic Surgeon | 1 |
|  | Internal Medicine with Cardiology | 2 |
| Received Formal PGx Education |  | 0 |

Table S4: Mapping of Emergent Subthemes to CFIR Constructs

| CFIR Construct | Mapped Subthemes |
| --- | --- |
| Intervention Characteristics | • Low perceived relevance in routine cardiology practice  • Perceived clinical value and benefits of PGx  • Cost constraints and insurance limitations  • Time constraints and incompatibility with acute care settings |
| Outer Setting | • Cost constraints and insurance limitations  • Demand for local guidelines and context-specific evidence  • Selective and condition-specific application of PGx |
| Inner Setting | • Absence of infrastructure and clinical workflow integration  • Time constraints and incompatibility with acute care settings |
| Characteristics of Individuals | • Limited clinical and educational exposure to PGx  • Low perceived relevance in practice  • Perceived clinical value and benefits of PGx |
| Process of Implementation | • Support for interdisciplinary implementation models  • Preference for simplified, physician-led integration  • Anticipated clinical value and future integration strategies |
