# Supplementary material for: Cardiologists’ perspectives on pharmacogenomics implementation in a hybrid health system: A qualitative study from the United Arab Emirates
Source: PLoS One. 2026 Jun 12;21(6):e0351218. doi: 10.1371/journal.pone.0351218 (PMC13262839; doi:10.1371/journal.pone.0351218)
Supplement: Attachment — Submitted filename: Response to Reviewer Cardiologists Study.docx [file pone.0351218.s002.docx]

Dear Dr Zawiah and Reviewers,

Thank you for your time and for your thoughtful, constructive comments on our manuscript. We have carefully considered all comments and have revised the manuscript to improve clarity, strengthen methodological transparency (particularly regarding data analysis, reflexivity, and saturation), and enhance the analytical depth of the findings..

Below, we provide a detailed, point-by-point response to each comment. All revisions have been incorporated into the manuscript and are highlighted in the revised version.

| Source | Comment | | Response |
| --- | --- | --- | --- |
| Journal Requirement | Please ensure that your manuscript meets PLOS ONE's style requirements, including those for file naming. | | The manuscript has been revised to fully comply with PLOS ONE formatting and file naming requirements. |
|  | We note that your ethics statement in the online submission form does not match the ethics statement in the methods section of your manuscript. | | The ethics approval number has been corrected and is now consistent across all sections of the manuscript. |
|  | Please note that your Data Availability Statement is currently missing the repository name and/or the DOI/accession number | | We have revised the Data Availability Statement to clarify that the data cannot be made publicly available due to ethical and confidentiality considerations, as the interviews contain potentially identifiable information. We now specify that data are available from the corresponding author upon reasonable request and with appropriate institutional approval. |
|  | Please ensure that you refer to Figure 1 in your text | | Figure 1 is now explicitly referenced in the main text. |
|  | Please include captions for your Supporting Information files at the end of your manuscript | | We have added a dedicated “Supporting Information” section at the end of the manuscript, including captions for all supplementary files and ensuring consistency with in-text citations. |
| Editor | Please clearly specify the qualitative study approach (e.g., phenomenological, descriptive, or other) | | We have revised the study design paragraph to clearly specify the qualitative approach. The study is now described as “a qualitative design with a descriptive orientation using an abductive analytical approach.” We further clarify that data were analysed inductively using thematic analysis, and the findings were subsequently mapped onto the Consolidated Framework for Implementation Research (CFIR) to support structured interpretation. This revision ensures methodological clarity and consistency with the analytical approach used. |
|  | Please add a dedicated paragraph clearly describing how trustworthiness was ensured | | A structured paragraph addressing credibility, dependability, confirmability, and transferability has been added. |
|  | Ensure consistency in ethical approval numbers. ERSC_2024_4212 (front section) ERSC_2024_4782 (methods) | | The ethical approval number has been corrected and is now consistent throughout the manuscript. |
| Reviewer #1 | please ensure terminology is consistent and defined at first use (EHR vs EMR; PGx vs pharmacogenomics; CDS as clinical decision support) | | We have revised the manuscript to ensure that all key terms are clearly defined at first use and used consistently throughout. Specifically, terminology related to pharmacogenomics (PGx), electronic health records (EHR), and clinical decision support (CDS) has been standardised across the manuscript. |
|  | The Introduction/Background would benefit from more clinically anchored examples of high-impact cardiovascular PGx applications | | We have revised the Introduction to include more clinically anchored examples of key pharmacogenomic applications in cardiovascular care. Specifically, we now provide examples of well-established gene–drug pairs, including CYP2C19–clopidogrel, CYP2C9/VKORC1–warfarin, and SLCO1B1–statins, along with their clinical implications. We have also included a brief reference to emerging evidence on beta-blocker response to further contextualise PGx relevance in cardiology. |
|  | the literature base is very dense; prioritizing the most directly supportive and preferably systematic/consensus evidence | | The Discussion has been revised to reduce redundancy and prioritize key evidence. |
|  | consider expanding the limitations to address the potential impact of online interviews… AI-assisted analytic steps… limited hands-on PGx experience | | We have expanded the limitations section to acknowledge that conducting interviews online may have limited the ability to capture non-verbal cues and may have influenced rapport during data collection.  We have clarified in the manuscript that the AI-assisted tool was used only to support the initial identification of preliminary codes. All subsequent coding, refinement, and thematic interpretation were conducted manually by the research team. Given this limited and supportive role, we did not consider it a substantive source of bias, but have ensured transparency in describing its use.  We have added a statement noting that most participants had limited formal training or hands-on experience in pharmacogenomics, which may have influenced their perspectives and affects the transferability of the findings to more established settings. |
| Reviewer #2 | The title is slightly long and dense; consider a brief title for the study. | | Changed to: “Cardiologists’ Perspectives on Pharmacogenomics Implementation in a Hybrid Health System: A Qualitative Study from the United Arab Emirates” |
|  | “Abductive study” may confuse the general readership. | | We have revised the wording in the abstract to improve clarity, replacing “abductive study” with “a qualitative study using an abductive analytical approach.” |
|  | The abstract lacks an explicit novelty statement | | We have revised the Background section of the abstract to more clearly articulate the study’s contribution by highlighting the limited understanding of how cardiologists perceive and navigate pharmacogenomics implementation within hybrid healthcare systems. |
|  | The introduction could be more analytical. There is a limitation of existing literature. | | We have revised the Introduction to strengthen its analytical depth by explicitly highlighting the limitations of existing literature. Specifically, we now emphasise the lack of real-world, clinician-level perspectives on pharmacogenomics implementation in cardiology within hybrid healthcare systems such as the UAE, which this study aims to address. |
|  | The research gap is not sharply framed. | | The research gap has been clarified and explicitly stated. |
|  | There are no qualitative CFIR-based studies given in the introduction in the UAE cardiology. | | We have revised the Introduction to more clearly articulate the research gap by explicitly highlighting the lack of context-specific, real-world evidence on pharmacogenomics implementation in cardiology within hybrid healthcare systems such as the UAE. |
|  | Reduce guideline listing (CPIC, DPWG, etc.) | | The guideline discussion has been streamlined. |
|  | The sampling strategy… introduces a risk of selection bias | We have addressed this in the limitations section by acknowledging that the use of purposive, convenience, and snowball sampling may introduce selection bias, including the possibility that participants were more interested in or positively inclined toward pharmacogenomics. | |
|  | Participants were more interested or positively inclined toward PGx | We have incorporated this point into the limitations, noting that participant inclination toward PGx may have influenced the views reported. | |
|  | limited detail on how saturation was assessed | We have revised the manuscript to provide a clearer description of how data saturation was assessed. Specifically, saturation was determined through concurrent coding, iterative comparison of emerging themes, and regular team discussions, with no new themes identified in the final interviews, indicating that meaning saturation had been achieved. | |
|  | Providing a clearer description of whether code or meaning saturation was reached | We have clarified in the manuscript that code saturation was achieved, as no new codes or themes were identified in the final interviews. We also describe how this was assessed through concurrent coding, iterative comparison of emerging themes, and regular team discussions. | |
|  | The use of an AI-based tool… not described | We have revised the Data Analysis section to clarify the role of the AI-assisted tool. The tool was used only to support the initial identification of preliminary codes. All outputs were critically reviewed, refined, and validated manually by the research team through an iterative coding process. The final coding structure and thematic interpretation were developed by the researchers to ensure analytical rigour and accuracy. | |
|  | there is no discussion of intercoder reliability | We have revised the Data Analysis section to provide greater detail on the coding process. A subset of transcripts was independently coded by two researchers, and discrepancies were discussed and resolved through consensus during regular team meetings. In line with qualitative research standards, we emphasised reflexive dialogue and consistency in interpretation rather than formal statistical measures of intercoder reliability | |
|  | Lacks sufficient detail on the researchers’ backgrounds, positionality | We have revised the manuscript to provide a clearer description of the interviewer’s background and role. Specifically, we now state that all interviews were conducted by a female PhD candidate in Public Health with a background in clinical pharmacy and formal training in qualitative research, who had no prior relationship with participants. We have also clarified how reflexivity was maintained through journaling and team discussions to reflect on potential influences on data collection and interpretation. | |
|  | The manuscript does not clearly explain how this mapping was conducted | We have clarified that themes were developed inductively and then mapped to CFIR constructs through an iterative, collaborative process. This mapping was reviewed within the research team to ensure conceptual alignment and consistency. A table is provided in the supplementary material for all details. | |
|  | Some themes remain largely descriptive | We have revised the Discussion to strengthen the analytical depth by incorporating additional interpretive statements that clarify the implications of key findings. Specifically, we have moved beyond descriptive reporting to better highlight how the findings inform understanding of implementation challenges across individual, organisational, and system levels. | |
|  | Inconsistencies in the ethical approval numbers | Corrected throughout the manuscript. | |
|  | The interview guide… should also be justified | We have added clarification that sharing the interview guide supported more considered and practice-based responses. Given the specialised nature of pharmacogenomics, this approach allowed participants to reflect on their real-world clinical experiences and provide structured, contextually grounded insights rather than purely spontaneous responses. | |
|  | Discussion is… somewhat lengthy and occasionally repetitive | The discussion has been streamlined and refined. | |
|  | Greater emphasis on the study’s theoretical contribution | We have strengthened the Discussion by adding a paragraph explicitly highlighting the study’s theoretical contribution. Specifically, we emphasise how the findings extend the application of the CFIR framework to a hybrid healthcare system and a cardiology-specific context, demonstrating how implementation factors interact across multiple levels. | |
|  | Some overlap between themes is evident | We recognise that some themes are conceptually related, reflecting the interconnected nature of implementation factors. We have clarified this in the Results section by indicating that themes are presented separately to reflect different levels of influence (individual, organisational, and system-level), while maintaining their conceptual relationships. | |
|  | figures… are informative but visually dense | We agree that the figures contain multiple elements due to the complexity of the CFIR framework and the multi-level nature of the findings. To improve clarity, we have revised the figure captions and corresponding text to better guide the reader and highlight key elements. We have retained the current level of detail to ensure that the full conceptual relationships are accurately represented. | |
