# Supplementary material for: Cardiologists’ perspectives on pharmacogenomics implementation in a hybrid health system: A qualitative study from the United Arab Emirates
Source: PLoS One. 2026 Jun 12;21(6):e0351218. doi: 10.1371/journal.pone.0351218 (PMC13262839; doi:10.1371/journal.pone.0351218)
Supplement: Peer review history

# Peer review: Decision Letter 0

**DOI:** 10.1371/journal.pone.0351218.r001

**Authors:** Mohammed Zawiah

18 Mar 2026

PONE-D-25-63908

Cardiologists’ Perspectives on the Feasibility of Implementing Pharmacogenomics in a Hybrid Health System: A CFIR-Guided Qualitative Study from the United Arab Emirates

PLOS One

Dear Dr. Al-Maskari,

Thank you for submitting your manuscript to PLOS ONE. After careful consideration, we feel that it has merit but does not fully meet PLOS ONE’s publication criteria as it currently stands. Therefore, we invite you to submit a revised version of the manuscript that addresses the points raised during the review process.

Please submit your revised manuscript by May 02 2026 11:59PM. If you will need more time than this to complete your revisions, please reply to this message or contact the journal office at plosone@plos.org. When you're ready to submit your revision, log on to https://www.editorialmanager.com/pone/ and select the 'Submissions Needing Revision' folder to locate your manuscript file.

Please include the following items when submitting your revised manuscript:

- A letter that responds to each point raised by the academic editor and reviewer(s). You should upload this letter as a separate file labeled 'Response to Reviewers'.
- A marked-up copy of your manuscript that highlights changes made to the original version. You should upload this as a separate file labeled 'Revised Manuscript with Track Changes'.
- An unmarked version of your revised paper without tracked changes. You should upload this as a separate file labeled 'Manuscript'.

If you would like to make changes to your financial disclosure, please include your updated statement in your cover letter. Guidelines for resubmitting your figure files are available below the reviewer comments at the end of this letter.

If applicable, we recommend that you deposit your laboratory protocols in protocols.io to enhance the reproducibility of your results. Protocols.io assigns your protocol its own identifier (DOI) so that it can be cited independently in the future. For instructions see: https://journals.plos.org/plosone/s/submission-guidelines#loc-laboratory-protocols. Additionally, PLOS ONE offers an option for publishing peer-reviewed Lab Protocol articles, which describe protocols hosted on protocols.io. Read more information on sharing protocols at https://plos.org/protocols?utm_medium=editorial-email&utm_source=authorletters&utm_campaign=protocols.

We look forward to receiving your revised manuscript.

Kind regards,

Mohammed Zawiah

Academic Editor

PLOS One

Journal Requirements:

When submitting your revision, we need you to address these additional requirements.

1.Please ensure that your manuscript meets PLOS ONE's style requirements, including those for file naming. The PLOS ONE style templates can be found at

https://journals.plos.org/plosone/s/file?id=wjVg/PLOSOne_formatting_sample_main_body.pdf and

https://journals.plos.org/plosone/s/file?id=ba62/PLOSOne_formatting_sample_title_authors_affiliations.pdf

2. We note that your ethics statement in the online submission form does not match the ethics statement in the methods section of your manuscript. When you resubmit please ensure the correct reference number for the approval is in both ethics statements.

3. Please note that your Data Availability Statement is currently missing the repository name and/or the DOI/accession number of each dataset OR a direct link to access each database. If your manuscript is accepted for publication, you will be asked to provide these details on a very short timeline. We therefore suggest that you provide this information now, though we will not hold up the peer review process if you are unable.

4. Please ensure that you refer to Figure 1 in your text as, if accepted, production will need this reference to link the reader to the figure.

5. Please include captions for your Supporting Information files at the end of your manuscript, and update any in-text citations to match accordingly. Please see our Supporting Information guidelines for more information: http://journals.plos.org/plosone/s/supporting-information.

6. If the reviewer comments include a recommendation to cite specific previously published works, please review and evaluate these publications to determine whether they are relevant and should be cited. There is no requirement to cite these works unless the editor has indicated otherwise.

Additional Editor Comments:

Dear Authors,

Thank you for submitting your manuscript. The manuscript addresses an important and timely topic with clear relevance to implementation science and precision medicine. The qualitative approach and use of CFIR provide a valuable framework for understanding system-level barriers and facilitators. However, several issues require clarification and revision before the manuscript can be considered for publication.

Please address the following key points:

1. Please clearly specify the qualitative study approach (e.g., phenomenological, descriptive, or other) in the study design paragraph, as this is currently not explicitly stated.

2. Please add a dedicated paragraph clearly describing how trustworthiness was ensured (e.g., credibility, dependability, confirmability, and transferability). While some elements (e.g., member checking, reflexivity) are mentioned, these should be explicitly consolidated and reported in a structured manner.

3. Ensure consistency in ethical approval numbers. ERSC_2024_4212 (front section) ERSC_2024_4782 (methods) !

In addition, please address the reviewers' comments alongside the points above.

Sincerely,

[Note: HTML markup is below. Please do not edit.]

Reviewer's Responses to Questions

**Comments to the Author**

1. Is the manuscript technically sound, and do the data support the conclusions?

The manuscript must describe a technically sound piece of scientific research with data that supports the conclusions. Experiments must have been conducted rigorously, with appropriate controls, replication, and sample sizes. The conclusions must be drawn appropriately based on the data presented.

Reviewer #1: Yes

Reviewer #2: Yes

**********

2. Has the statistical analysis been performed appropriately and rigorously?

Reviewer #1: N/A

Reviewer #2: Yes

**********

3. Have the authors made all data underlying the findings in their manuscript fully available?

The PLOS Data policy requires authors to make all data underlying the findings described in their manuscript fully available without restriction, with rare exception (please refer to the Data Availability Statement in the manuscript PDF file). The data should be provided as part of the manuscript or its supporting information, or deposited to a public repository. For example, in addition to summary statistics, the data points behind means, medians and variance measures should be available. If there are restrictions on publicly sharing data—e.g. participant privacy or use of data from a third party—those must be specified.

Reviewer #1: Yes

Reviewer #2: Yes

**********

4. Is the manuscript presented in an intelligible fashion and written in standard English?

PLOS ONE does not copyedit accepted manuscripts, so the language in submitted articles must be clear, correct, and unambiguous. Any typographical or grammatical errors should be corrected at revision, so please note any specific errors here.

Reviewer #1: Yes

Reviewer #2: Yes

**********

5. Review Comments to the Author

Please use the space provided to explain your answers to the questions above. You may also include additional comments for the author, including concerns about dual publication, research ethics, or publication ethics. (Please upload your review as an attachment if it exceeds 20,000 characters)

Reviewer #1: Here are some suggestions for enhancing the quality of manuscript: please ensure terminology is consistent and defined at first use (EHR vs EMR; PGx vs pharmacogenomics; CDS as clinical decision support), and then applied uniformly throughout the manuscript. The Introduction/Background would benefit from more clinically anchored examples of high-impact cardiovascular PGx applications (e.g., clopidogrel–CYP2C19, warfarin–CYP2C9/VKORC1, statins–SLCO1B1) with a brief justification for their priority in the UAE context. In the Discussion, the literature base is very dense; prioritizing the most directly supportive and preferably systematic/consensus evidence and removing redundant citations would improve narrative focus. Finally, consider expanding the limitations to address the potential impact of online interviews on rapport and non-verbal cues, potential bias introduced by any AI-assisted analytic steps, and the implications of limited hands-on PGx experience among participants for transferability/generalizability.

Reviewer #2: The manuscript addresses an important and timely topic, exploring cardiologists’ perspectives on pharmacogenomics implementation within a hybrid healthcare system using a CFIR-guided qualitative approach. The study is relevant, well-structured, and offers meaningful insights with practical implications. However, several methodological and reporting aspects require clarification and strengthening.

Title

1. The title is slightly long and dense; consider a brief title for the study.

Abstract

1. “Abductive study” may confuse the general readership.

2. The abstract lacks an explicit novelty statement.

Introduction

1. The introduction could be more analytical. There is a limitation of existing literature. Add not just “what is known” but “what is missing.”

2. The research gap is not sharply framed.

3. There are no qualitative CFIR-based studies given in the introduction in the UAE cardiology.

4. Reduce guideline listing (CPIC, DPWG, etc.)

Methodology

1. The sampling strategy, which combines purposive, convenience, and snowball approaches, introduces a risk of selection bias that is not sufficiently acknowledged or justified.

2. While qualitative studies do not aim for statistical generalizability, the authors should more explicitly discuss how this approach may have influenced the perspectives captured, particularly the possibility that participants were more interested or positively inclined toward PGx.

3. Although the authors state that data saturation was achieved, there is limited detail on how saturation was assessed or operationalized.

4. Providing a clearer description of whether code or meaning saturation was reached, and at what stage, would enhance methodological credibility.

5. The data analysis section would also benefit from greater transparency. The use of an AI-based tool to generate preliminary codes is mentioned, but the specific tool, its role, and how its outputs were validated are not described.

6. Additionally, there is no discussion of intercoder reliability or how disagreements were resolved during coding, which is important for ensuring analytic rigor in qualitative research. While reflexivity is briefly acknowledged, the manuscript lacks sufficient detail on the researchers’ backgrounds, positionality, and potential influence on data interpretation, which is a key component of qualitative methodology.

7. Another area of concern relates to the application of CFIR. Although the abductive approach, where themes were developed inductively and later mapped onto CFIR, is appropriate, the manuscript does not clearly explain how this mapping was conducted or whether it was independently validated.

8. In addition, while the findings are well presented, some themes remain largely descriptive, and the discussion could be enhanced by deeper critical engagement with the data, particularly in terms of how the findings extend or challenge existing implementation science literature.

9. From a reporting perspective, there are inconsistencies in the ethical approval numbers presented in different sections of the manuscript, which should be corrected for accuracy.

10. The decision to share the interview guide with participants in advance should also be justified, as this may have influenced the spontaneity of responses.

Results

1. The results section is generally well organized and supported by illustrative quotations, which enhance credibility; however, some overlap between themes is evident, and further refinement could improve clarity.

2. The figures, particularly the CFIR mapping diagram, are informative but visually dense and may benefit from simplification.

Discussion

1. The discussion is comprehensive and effectively situates the findings within the global literature, but it is somewhat lengthy and occasionally repetitive, especially regarding barriers, reiterating results rather than advancing interpretation.

2. Greater emphasis on the study’s theoretical contribution and comparison with similar settings, including low- and middle-income contexts, would strengthen the manuscript’s broader relevance.

**********

6. PLOS authors have the option to publish the peer review history of their article (what does this mean?). If published, this will include your full peer review and any attached files.

If you choose “no”, your identity will remain anonymous but your review may still be made public.

**Do you want your identity to be public for this peer review?**   For information about this choice, including consent withdrawal, please see our Privacy Policy.

Reviewer #1: No

Reviewer #2: **Yes:**   Rabbiya Ahmad

**********

[NOTE: If reviewer comments were submitted as an attachment file, they will be attached to this email and accessible via the submission site. Please log into your account, locate the manuscript record, and check for the action link "View Attachments". If this link does not appear, there are no attachment files.]

To ensure your figures meet our technical requirements, please review our figure guidelines: https://journals.plos.org/plosone/s/figures

You may also use PLOS’s free figure tool, NAAS, to help you prepare publication quality figures: https://journals.plos.org/plosone/s/figures#loc-tools-for-figure-preparation.

NAAS will assess whether your figures meet our technical requirements by comparing each figure against our figure specifications.

# Author comment: Author response to Decision Letter 1

**DOI:** 10.1371/journal.pone.0351218.r002

20 Apr 2026

Dear Dr Zawiah and Reviewers,

Thank you for your time and for your thoughtful, constructive comments on our manuscript. We have carefully considered all comments and have revised the manuscript to improve clarity, strengthen methodological transparency (particularly regarding data analysis, reflexivity, and saturation), and enhance the analytical depth of the findings..

Below, we provide a detailed, point-by-point response to each comment. All revisions have been incorporated into the manuscript and are highlighted in the revised version.

Source Comment Response

Journal Requirement Please ensure that your manuscript meets PLOS ONE's style requirements, including those for file naming. The manuscript has been revised to fully comply with PLOS ONE formatting and file naming requirements.

We note that your ethics statement in the online submission form does not match the ethics statement in the methods section of your manuscript. The ethics approval number has been corrected and is now consistent across all sections of the manuscript.

Please note that your Data Availability Statement is currently missing the repository name and/or the DOI/accession number We have revised the Data Availability Statement to clarify that the data cannot be made publicly available due to ethical and confidentiality considerations, as the interviews contain potentially identifiable information. We now specify that data are available from the corresponding author upon reasonable request and with appropriate institutional approval.

Please ensure that you refer to Figure 1 in your text Figure 1 is now explicitly referenced in the main text.

Please include captions for your Supporting Information files at the end of your manuscript We have added a dedicated “Supporting Information” section at the end of the manuscript, including captions for all supplementary files and ensuring consistency with in-text citations.

Editor Please clearly specify the qualitative study approach (e.g., phenomenological, descriptive, or other) We have revised the study design paragraph to clearly specify the qualitative approach. The study is now described as “a qualitative design with a descriptive orientation using an abductive analytical approach.” We further clarify that data were analysed inductively using thematic analysis, and the findings were subsequently mapped onto the Consolidated Framework for Implementation Research (CFIR) to support structured interpretation. This revision ensures methodological clarity and consistency with the analytical approach used.

Please add a dedicated paragraph clearly describing how trustworthiness was ensured A structured paragraph addressing credibility, dependability, confirmability, and transferability has been added.

Ensure consistency in ethical approval numbers. ERSC_2024_4212 (front section) ERSC_2024_4782 (methods) The ethical approval number has been corrected and is now consistent throughout the manuscript.

Reviewer #1 please ensure terminology is consistent and defined at first use (EHR vs EMR; PGx vs pharmacogenomics; CDS as clinical decision support) We have revised the manuscript to ensure that all key terms are clearly defined at first use and used consistently throughout. Specifically, terminology related to pharmacogenomics (PGx), electronic health records (EHR), and clinical decision support (CDS) has been standardised across the manuscript.

The Introduction/Background would benefit from more clinically anchored examples of high-impact cardiovascular PGx applications We have revised the Introduction to include more clinically anchored examples of key pharmacogenomic applications in cardiovascular care. Specifically, we now provide examples of well-established gene–drug pairs, including CYP2C19–clopidogrel, CYP2C9/VKORC1–warfarin, and SLCO1B1–statins, along with their clinical implications. We have also included a brief reference to emerging evidence on beta-blocker response to further contextualise PGx relevance in cardiology.

the literature base is very dense; prioritizing the most directly supportive and preferably systematic/consensus evidence The Discussion has been revised to reduce redundancy and prioritize key evidence.

consider expanding the limitations to address the potential impact of online interviews… AI-assisted analytic steps… limited hands-on PGx experience We have expanded the limitations section to acknowledge that conducting interviews online may have limited the ability to capture non-verbal cues and may have influenced rapport during data collection.

We have clarified in the manuscript that the AI-assisted tool was used only to support the initial identification of preliminary codes. All subsequent coding, refinement, and thematic interpretation were conducted manually by the research team. Given this limited and supportive role, we did not consider it a substantive source of bias, but have ensured transparency in describing its use.

We have added a statement noting that most participants had limited formal training or hands-on experience in pharmacogenomics, which may have influenced their perspectives and affects the transferability of the findings to more established settings.

Reviewer #2 The title is slightly long and dense; consider a brief title for the study. Changed to: “Cardiologists’ Perspectives on Pharmacogenomics Implementation in a Hybrid Health System: A Qualitative Study from the United Arab Emirates”

“Abductive study” may confuse the general readership. We have revised the wording in the abstract to improve clarity, replacing “abductive study” with “a qualitative study using an abductive analytical approach.”

The abstract lacks an explicit novelty statement We have revised the Background section of the abstract to more clearly articulate the study’s contribution by highlighting the limited understanding of how cardiologists perceive and navigate pharmacogenomics implementation within hybrid healthcare systems.

The introduction could be more analytical. There is a limitation of existing literature. We have revised the Introduction to strengthen its analytical depth by explicitly highlighting the limitations of existing literature. Specifically, we now emphasise the lack of real-world, clinician-level perspectives on pharmacogenomics implementation in cardiology within hybrid healthcare systems such as the UAE, which this study aims to address.

The research gap is not sharply framed. The research gap has been clarified and explicitly stated.

There are no qualitative CFIR-based studies given in the introduction in the UAE cardiology. We have revised the Introduction to more clearly articulate the research gap by explicitly highlighting the lack of context-specific, real-world evidence on pharmacogenomics implementation in cardiology within hybrid healthcare systems such as the UAE.

Reduce guideline listing (CPIC, DPWG, etc.) The guideline discussion has been streamlined.

The sampling strategy… introduces a risk of selection bias We have addressed this in the limitations section by acknowledging that the use of purposive, convenience, and snowball sampling may introduce selection bias, including the possibility that participants were more interested in or positively inclined toward pharmacogenomics.

Participants were more interested or positively inclined toward PGx We have incorporated this point into the limitations, noting that participant inclination toward PGx may have influenced the views reported.

limited detail on how saturation was assessed We have revised the manuscript to provide a clearer description of how data saturation was assessed. Specifically, saturation was determined through concurrent coding, iterative comparison of emerging themes, and regular team discussions, with no new themes identified in the final interviews, indicating that meaning saturation had been achieved.

Providing a clearer description of whether code or meaning saturation was reached We have clarified in the manuscript that code saturation was achieved, as no new codes or themes were identified in the final interviews. We also describe how this was assessed through concurrent coding, iterative comparison of emerging themes, and regular team discussions.

The use of an AI-based tool… not described We have revised the Data Analysis section to clarify the role of the AI-assisted tool. The tool was used only to support the initial identification of preliminary codes. All outputs were critically reviewed, refined, and validated manually by the research team through an iterative coding process. The final coding structure and thematic interpretation were developed by the researchers to ensure analytical rigour and accuracy.

there is no discussion of intercoder reliability We have revised the Data Analysis section to provide greater detail on the coding process. A subset of transcripts was independently coded by two researchers, and discrepancies were discussed and resolved through consensus during regular team meetings. In line with qualitative research standards, we emphasised reflexive dialogue and consistency in interpretation rather than formal statistical measures of intercoder reliability

Lacks sufficient detail on the researchers’ backgrounds, positionality We have revised the manuscript to provide a clearer description of the interviewer’s background and role. Specifically, we now state that all interviews were conducted by a female PhD candidate in Public Health with a background in clinical pharmacy and formal training in qualitative research, who had no prior relationship with participants. We have also clarified how reflexivity was maintained through journaling and team discussions to reflect on potential influences on data collection and interpretation.

The manuscript does not clearly explain how this mapping was conducted We have clarified that themes were developed inductively and then mapped to CFIR constructs through an iterative, collaborative process. This mapping was reviewed within the research team to ensure conceptual alignment and consistency. A table is provided in the supplementary material for all details.

Some themes remain largely descriptive We have revised the Discussion to strengthen the analytical depth by incorporating additional interpretive statements that clarify the implications of key findings. Specifically, we have moved beyond descriptive reporting to better highlight how the findings inform understanding of implementation challenges across individual, organisational, and system levels.

Inconsistencies in the ethical approval numbers Corrected throughout the manuscript.

The interview guide… should also be justified We have added clarification that sharing the interview guide supported more considered and practice-based responses. Given the specialised nature of pharmacogenomics, this approach allowed participants to reflect on their real-world clinical experiences and provide structured, contextually grounded insights rather than purely spontaneous responses.

Discussion is… somewhat lengthy and occasionally repetitive The discussion has been streamlined and refined.

Greater emphasis on the study’s theoretical contribution We have strengthened the Discussion by adding a paragraph explicitly highlighting the study’s theoretical contribution. Specifically, we emphasise how the findings extend the application of the CFIR framework to a hybrid healthcare system and a cardiology-specific context, demonstrating how implementation factors interact across multiple levels.

Some overlap between themes is evident We recognise that some themes are conceptually related, reflecting the interconnected nature of implementation factors. We have clarified this in the Results section by indicating that themes are presented separately to reflect different levels of influence (individual, organisational, and system-level), while maintaining their conceptual relationships.

figures… are informative but visually dense We agree that the figures contain multiple elements due to the complexity of the CFIR framework and the multi-level nature of the findings. To improve clarity, we have revised the figure captions and corresponding text to better guide the reader and highlight key elements. We have retained the current level of detail to ensure that the full conceptual relationships are accurately represented.

Attachment Submitted filename: Response to Reviewer Cardiologists Study.docx

# Peer review: Decision Letter 1

**DOI:** 10.1371/journal.pone.0351218.r003

**Authors:** Mohammed Zawiah

26 May 2026

"Cardiologists’ Perspectives on Pharmacogenomics Implementation in a Hybrid Health System: A Qualitative Study from the United Arab Emirates

PONE-D-25-63908R1

Dear Dr. Al-Maskari,

We’re pleased to inform you that your manuscript has been judged scientifically suitable for publication and will be formally accepted for publication once it meets all outstanding technical requirements.

Within one week, you’ll receive an e-mail detailing the required amendments. When these have been addressed, you’ll receive a formal acceptance letter and your manuscript will be scheduled for publication.

An invoice will be generated when your article is formally accepted. Please note, if your institution has a publishing partnership with PLOS and your article meets the relevant criteria, all or part of your publication costs will be covered. Please make sure your user information is up-to-date by logging into Editorial Manager at Editorial Manager® and clicking the ‘Update My Information' link at the top of the page. For questions related to billing, please contact billing support.

If your institution or institutions have a press office, please notify them about your upcoming paper to help maximize its impact. If they’ll be preparing press materials, please inform our press team as soon as possible -- no later than 48 hours after receiving the formal acceptance. Your manuscript will remain under strict press embargo until 2 pm Eastern Time on the date of publication. For more information, please contact onepress@plos.org.

Kind regards,

Mohammed Zawiah

Academic Editor

PLOS One

Additional Editor Comments (optional):

Reviewers' comments:

Reviewer's Responses to Questions

**Comments to the Author**

1. If the authors have adequately addressed your comments raised in a previous round of review and you feel that this manuscript is now acceptable for publication, you may indicate that here to bypass the “Comments to the Author” section, enter your conflict of interest statement in the “Confidential to Editor” section, and submit your "Accept" recommendation.

Reviewer #1: All comments have been addressed

**********

2. Is the manuscript technically sound, and do the data support the conclusions?

The manuscript must describe a technically sound piece of scientific research with data that supports the conclusions. Experiments must have been conducted rigorously, with appropriate controls, replication, and sample sizes. The conclusions must be drawn appropriately based on the data presented.

Reviewer #1: Yes

**********

3. Has the statistical analysis been performed appropriately and rigorously?

Reviewer #1: Yes

**********

4. Have the authors made all data underlying the findings in their manuscript fully available?

The PLOS Data policy requires authors to make all data underlying the findings described in their manuscript fully available without restriction, with rare exception (please refer to the Data Availability Statement in the manuscript PDF file). The data should be provided as part of the manuscript or its supporting information, or deposited to a public repository. For example, in addition to summary statistics, the data points behind means, medians and variance measures should be available. If there are restrictions on publicly sharing data—e.g. participant privacy or use of data from a third party—those must be specified.

Reviewer #1: Yes

**********

5. Is the manuscript presented in an intelligible fashion and written in standard English?

PLOS ONE does not copyedit accepted manuscripts, so the language in submitted articles must be clear, correct, and unambiguous. Any typographical or grammatical errors should be corrected at revision, so please note any specific errors here.

Reviewer #1: Yes

**********

6. Review Comments to the Author

Please use the space provided to explain your answers to the questions above. You may also include additional comments for the author, including concerns about dual publication, research ethics, or publication ethics. (Please upload your review as an attachment if it exceeds 20,000 characters)

Reviewer #1: No more comments. I dont have concerns about dual publication, research ethics, or publication ethics.

**********

7. PLOS authors have the option to publish the peer review history of their article (what does this mean?). If published, this will include your full peer review and any attached files.

If you choose “no”, your identity will remain anonymous but your review may still be made public.

**Do you want your identity to be public for this peer review?**   For information about this choice, including consent withdrawal, please see our Privacy Policy.

Reviewer #1: **Yes:**   Liangkun Guo

**********

# Editor report: Acceptance letter

**DOI:** 10.1371/journal.pone.0351218.r004

**Authors:** Mohammed Zawiah

PONE-D-25-63908R1

PLOS One

Dear Dr. Al-Maskari,

I'm pleased to inform you that your manuscript has been deemed suitable for publication in PLOS One. Congratulations! Your manuscript is now being handed over to our production team.

At this stage, our production department will prepare your paper for publication. This includes ensuring the following:

* All references, tables, and figures are properly cited

* All relevant supporting information is included in the manuscript submission,

* There are no issues that prevent the paper from being properly typeset

You will receive further instructions from the production team, including instructions on how to review your proof when it is ready. Please keep in mind that we are working through a large volume of accepted articles, so please give us a few days to review your paper and let you know the next and final steps.

Lastly, if your institution or institutions have a press office, please let them know about your upcoming paper now to help maximize its impact. If they'll be preparing press materials, please inform our press team within the next 48 hours. Your manuscript will remain under strict press embargo until 2 pm Eastern Time on the date of publication. For more information, please contact onepress@plos.org.

You will receive an invoice from PLOS for your publication fee after your manuscript has reached the completed accept phase. If you receive an email requesting payment before acceptance or for any other service, this may be a phishing scheme. Learn how to identify phishing emails and protect your accounts at https://explore.plos.org/phishing.

If we can help with anything else, please email us at customercare@plos.org.

Thank you for submitting your work to PLOS ONE and supporting open access.

Kind regards,

PLOS ONE Editorial Office Staff

on behalf of

Dr. Mohammed Zawiah

Academic Editor

PLOS One
